# A Scoping Review of Professional Identity Formation in Undergraduate Medical Education

**DOI:** 10.1007/s11606-021-07024-9

**Published:** 2021-08-16

**Authors:** Shiva Sarraf-Yazdi, Yao Neng Teo, Ashley Ern Hui How, Yao Hao Teo, Sherill Goh, Cheryl Shumin Kow, Wei Yi Lam, Ruth Si Man Wong, Haziratul Zakirah Binte Ghazali, Sarah-Kei Lauw, Javier Rui Ming Tan, Ryan Bing Qian Lee, Yun Ting Ong, Natalie Pei Xin Chan, Clarissa Wei Shuen Cheong, Nur Haidah Ahmad Kamal, Alexia Sze Inn Lee, Lorraine Hui En Tan, Annelissa Mien Chew Chin, Min Chiam, Lalit Kumar Radha Krishna

**Affiliations:** 1 Duke-NUS Medical School, Singapore, Singapore; 2 Yong Loo Lin School of Medicine, National University of Singapore, Singapore, Singapore; 3 Division of Supportive and Palliative Care, National Cancer Centre Singapore, Singapore, Singapore; 4 Lee Kong Chian School of Medicine, Nanyang Technological University, Singapore, Singapore; 5 Singapore Health Services, Singapore, Singapore; 6 Division of Cancer Education, National Cancer Centre Singapore, Singapore, Singapore; 7 Medical Library, National University of Singapore Libraries, Singapore, Singapore; 8 Palliative Care Institute Liverpool, Academic Palliative & End of Life Care Centre, University of Liverpool, United Kingdom, Cancer Research Centre, University of Liverpool, Liverpool, UK; 9 Centre of Biomedical Ethics, National University of Singapore, Singapore, Singapore; 10 PalC, The Palliative Care Centre for Excellence in Research and Education, Singapore, Singapore

**Keywords:** professional identity, professional identity formation, PIF, personhood, ring theory of personhood

## Abstract

**Background:**

Professional identity formation (PIF) in medical students is a multifactorial phenomenon, shaped by ways that clinical and non-clinical experiences, expectations and environmental factors merge with individual values, beliefs and obligations. The relationship between students’ evolving professional identity and self-identity or personhood remains ill-defined, making it challenging for medical schools to support PIF systematically and strategically. Primarily, to capture prevailing literature on PIF in medical school education, and secondarily, to ascertain how PIF influences on medical students may be viewed through the lens of the ring theory of personhood (RToP) and to identify ways that medical schools support PIF.

**Methods:**

A systematic scoping review was conducted using the systematic evidence-based approach. Articles published between 1 January 2000 and 1 July 2020 related to PIF in medical students were searched using PubMed, Embase, PsycINFO, ERIC and Scopus. Articles of all study designs (quantitative and qualitative), published or translated into English, were included. Concurrent thematic and directed content analyses were used to evaluate the data.

**Results:**

A total of 10443 abstracts were identified, 272 full-text articles evaluated, and 76 articles included. Thematic and directed content analyses revealed similar themes and categories as follows: characteristics of PIF in relation to professionalism, role of socialization in PIF, PIF enablers and barriers, and medical school approaches to supporting PIF.

**Discussion:**

PIF involves iterative construction, deconstruction and inculcation of professional beliefs, values and behaviours into a pre-existent identity. Through the lens of RToP, factors were elucidated that promote or hinder students’ identity development on individual, relational or societal levels. If inadequately or inappropriately supported, enabling factors become barriers to PIF. Medical schools employ an all-encompassing approach to support PIF, illuminating the need for distinct and deliberate longitudinal monitoring and mentoring to foster students’ balanced integration of personal and professional identities over time.

**Supplementary Information:**

The online version contains supplementary material available at 10.1007/s11606-021-07024-9.

## INTRODUCTION

Professional identity in medicine refers to one’s “interpretation of what being a good doctor means and the manner in which he or she should behave” ^1^. Holden et al. ^2^ describe professional identity formation (PIF) “as the foundational process one experiences during the transformation from lay person to physician”. Growing data suggest that PIF is heavily influenced by how medical students evaluate their professional roles and responsibilities in light of fluid circumstances and clinical experiences. This developmental process is shaped by sociocultural, familial, academic, moral, religious and gender-based roles, values, beliefs and obligations ^3–6^. The complexity herein underlines the challenge that medical schools face in viewing and reviewing their approaches to fostering PIF ^7–10^.

Identity is a manifestation of qualities, conditions, beliefs, values and ideals that humans possess and regard with importance. While core components remain foundational and enduring, identity exists in a perpetual state of flux with elements taking on different forms and priorities. Moss et al.^11^ posit that professional identity “is the integration of the professional self and the personal self”. This suggests a connection between PIF in medical school and the students’ own concept of identity or personhood.

Personhood has been conceived in a plethora of ways. While Buron’s ^12^ levels of personhood considers individual, biological and sociological concepts, Dennett ^13^ underscores the importance of communicative and cognitive faculties. A number of these concepts incorporate Lockean ^14^ and Kantian’s ^15^ formulations that necessitate the presence of consciousness, rationality, self-awareness, intelligence, moral value, attainment of legal status ^16^ and personal, enduring interests ^17–20^. What these static frameworks do not consider is the dynamic influence of one’s changing beliefs, attitudes and perceptions on decision-making ^21–23^. Similarly, existing concepts of PIF in medical students do not holistically acknowledge the evolving *person* behind the budding *professional*.

**Figure 1:**
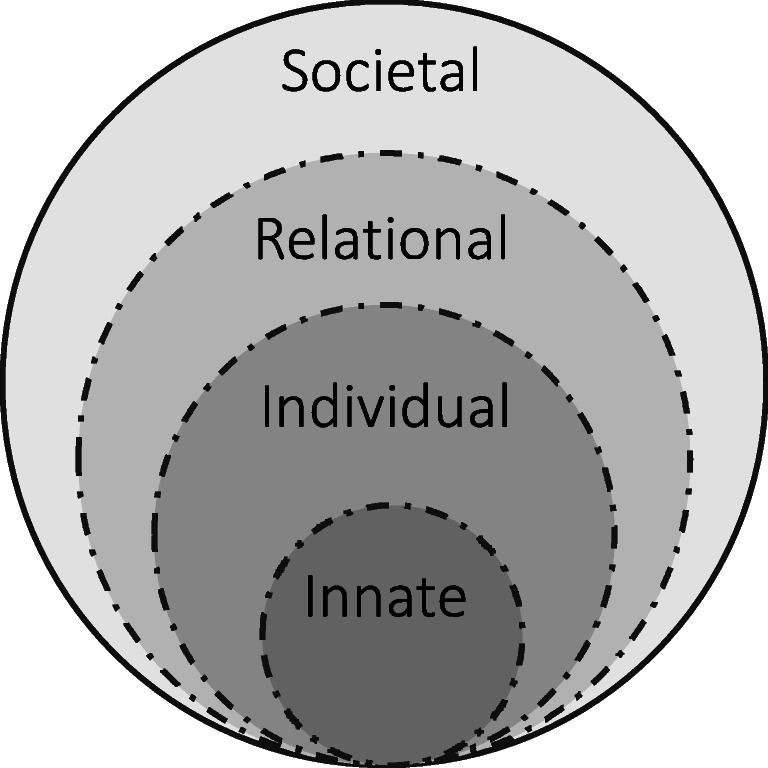
The four rings of personhood in RToP

To explore these gaps, we adopted Krishna and Alsuwaigh’s ring theory of personhood (RToP) ^24,25^, which characterizes personhood as four interconnected rings — the Innate, Individual, Relational and Societal (Figure 1). This framework considers the evolving nature of personhood and various sources of influence that inform one’s self-concept of identity, i.e. what makes us who we are ^26,27^. The *Innate* Ring represents qualities that remain steadfast such as an individual’s genetic makeup and the family, society, culture, religion, race and gender into which an individual is born. Though some features may change, these impact an individual’s development and often form the basis of who they are as a person. The *Individual* Ring represents one’s conscious function and ability to communicate and display emotions. Beliefs and values within this ring are informed by its specific contents. A religious individual, for example, holds beliefs, values and principles associated with their religious stance. The more strongly the individual upholds these, the more it impacts their thoughts, decisions and actions. This highlights the entwined nature of various aspects of personhood and the role of the Individual Ring in shaping identity. The *Relational* Ring depicts the close personal ties that one shares with those deemed important. The *Societal* Ring houses more distant relationships as well as social expectations, cultural norms, professional standards and religious obligations placed upon the individual. These include codes of conduct and practice expected of the person by virtue of their membership within society.

One’s self-concept of identity can thus influence, exist as a part of, and encapsulate an evolving professional identity. To explore this concept in the medical school context, we aimed to capture the various elements of PIF through a scoping review, and used the RToP as an organizing framework to explore how fluid circumstances related to professional identity development may affect a medical student’s personhood.

## METHODS

**Figure 2:**
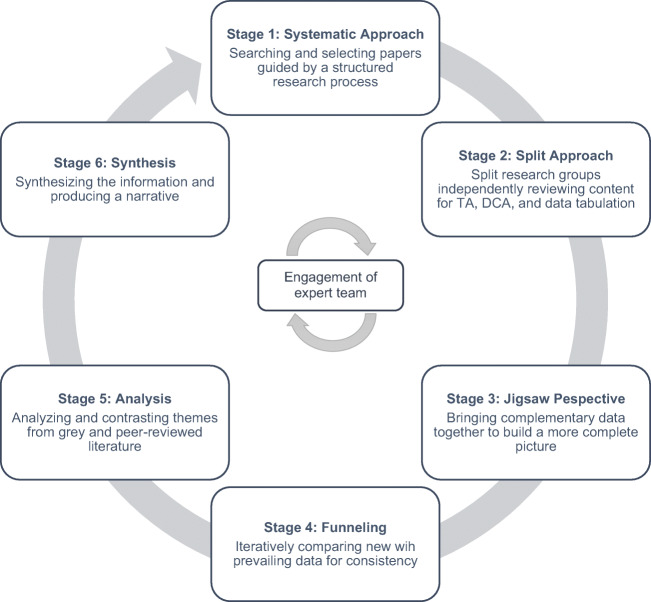
A schematic of the steps involved in systematic evidence-based approach (SEBA). Abbreviations: TA, thematic analysis; DCA, directed content analysis; BEME, Best Evidence Medical Education; STORIES, Structured approach to the Reporting In healthcare education of Evidence Synthesis

We used a systematic scoping review (SSR) to map available data on PIF in prevailing undergraduate medical education literature and to identify information related to key characteristics of PIF within this context ^28,29^. To overcome the absence of a consistent approach to conducting scoping reviews ^30^, a 16-member research team applied Krishna’s systematic evidence-based approach (SEBA) ^31–33^. The six-stage structured process (Figure 2) provides a reproducible and transparent means of reviewing the search process, and the manner in which the data was accrued, analyzed and used to inform the conclusions drawn within the SSR.

SEBA’s constructivist perspective allowed for capture of psychosocial, cultural and historical influences that underpin individual concepts of PIF, and its relativist lens enabled a holistic picture by considering various perspectives through data collected from quantitative, qualitative and knowledge synthesis articles.

Each stage of SEBA additionally involved input from an expert team that guided the practical approach to the project, while independently reviewing and accounting for data collection, analysis and synthesis. The expert team comprised a medical librarian from the Yong Loo Lin School of Medicine (YLLSoM) at the National University of Singapore (NUS), and educational experts and clinicians from the National Cancer Centre Singapore (NCCS), the Palliative Care Institute Liverpool, YLLSoM and Duke-NUS Medical School.

### Stage 1: Systematic Approach

A. Determining the background of review

The research and expert teams reviewed the overall objectives of the SSR, and determined the population, context and concept to be evaluated. This decision was guided by the preferred reporting items for systematic review and meta-analysis protocols (PRISMA-P) 2015 checklist ^34,35^ (see *Appendix* *1*).

B. Identifying research questions and Inclusion Criteria

Teams agreed for the primary research question to be “what is known of PIF in medical school education?” To ascertain the wider impact of PIF on the self-concept of medical students, these secondary research questions were identified: “how may influences of PIF be viewed through the RTOP lens?” and “how do medical schools support PIF?”

A PICOS format framed the research process ^36,37^ and may be found in *Appendix* *2*. Guided by the expert team and prevailing descriptions of PIF, the research team developed a search strategy for PubMed, Embase, PsycINFO, ERIC and Scopus databases. Independent searches were carried out for articles published between 1 January 2000 and 1 July 2020. The full PubMed search strategy may be found in *Appendix* *3*. All research methodologies (quantitative and qualitative) in articles published or translated into English were included.

III. Selecting included articles

The sixteen members of the research team independently reviewed the identified titles and abstracts, created lists of articles to be included, discussed these online, and reached consensus using Sandelowski and Barroso’s ^38^ “negotiated consensual validation” approach. Acknowledging limitations of the search terms, the members also performed reference snowballing. The PRISMA flow diagram can be found in *Appendix* *4*.

IV. Assessing quality of articles

Eight research team members individually appraised the quality of the quantitative and qualitative studies using the Medical Education Research Study Quality Instrument (MERSQI) ^39^ and Consolidated Criteria for Reporting Qualitative Studies (COREQ) ^40^. This allowed us to evaluate the methodology employed in the included articles, aid readers and reviewers in appraising the extent to which we reported the data, the weight we afforded the data in our analysis ^41^ and assist decision-makers in understanding the transferability of the findings ^42^. The analysis of 43 of 76 included articles amenable to quality appraisals may be found in *Appendix* *5*.

### Stage 2: Split Approach

To increase the reliability and transparency of the analysis, the Split Approach was adopted ^43,44^. Seven members of the research team independently analyzed the data using Braun and Clarke’s ^45^ approach to *thematic analysis*. Concurrently, nine members of the research team employed Hsieh and Shannon’s^46^ *directed content analysis* to independently analyze the data. This concurrent analysis aimed to reduce omission of new findings or negative reports and enable review of data from different perspectives. The reviewers within each sub-team achieved consensus on their analyses before comparing with the other two.

A. Thematic analysis

In the absence of rigorous definitions of PIF, seven members of the research team adopted Braun and Clarke’s approach to identifying key themes across different learning settings and learner/instructor populations ^47,48^. This allowed for analysis of data derived from quantitative, qualitative and mixed methodologies ^49,50^. This sub-team independently reviewed the included articles, constructed codes from surface meaning of the text and collated these into a code book, which was used to code and analyze the rest of the articles in an iterative process. New codes were associated with prior codes and concepts ^51,52^. An inductive approach allowed us to identify codes and themes from the raw data without using existing frameworks or preconceived notions as to how the data should be organized. The sub-team discussed their independent analyses in online and face-to-face meetings and used “negotiated consensual validation” to derive the final themes.

B. Directed content analysis

Nine members of the research team independently employed Hsieh and Shannon’s approach to directed content analysis. This involved “identifying and operationalizing *a priori* coding categories” by classifying text of similar meaning into categories drawn from prevailing theories ^46^. Four members first used deductive category application ^53^ to extract codes and categories from Cruess et al.’s^54^ article, “A schematic representation of the professional identity formation and socialization of medical students and residents: A guide for medical educators”.

Separately, to ensure adequate focus on the RToP domains, five members used Krishna and Alsuwaigh’s^24^ article, “Understanding the fluid nature of personhood – the ring theory of personhood” to draw the categories to be used as part of Hsieh and Shannon’s approach to directed content analysis. This was to evaluate the prevailing data through the lens of RToP and to answer the secondary research question on how PIF influences may be viewed through the lens of RToP. A code book was developed and individual findings were discussed through online and face-to-face meetings. Differences in codes were resolved until consensus was achieved on a final list of categories.

### Stage 3: Jigsaw Perspective

The Jigsaw Perspective hinges on Moss and Haertel’s^55^ suggestion that complementary qualitative data should be reviewed together to give “a richer, more nuanced understanding of a given phenomenon”. It considers each finding as a piece of jigsaw that combined with appropriate or complementary pieces, portrays a more complete picture. The research team thus identified and combined significant overlaps and similarities between themes and categories to gather a holistic picture of available data on PIF and RToP.

### Stage 4: Funnelling

Six members of the research team further summarized and tabulated the full-text articles included in the review according to Wong et al.’s^56^ RAMESES publication standards and Popay et al.’s^57^ guide to conducting narrative synthesis in systematic reviews. This was to verify that the jigsaw pieces appropriately reflected key insights from the prevailing data, ensuring that critical information was not lost.

To assist with this process, the team adopted Phases 3 to 6 of France et al.’s^58^ adaptation of Noblit and Hare’s ^59^ seven phases of meta-ethnography to study the included articles ^60^. In line with Phase 3, the study aim, key findings and insights were included in the tabulated summaries. In line with Phase 4, the team juxtaposed the themes and categories by grouping them, guided by the commensurate focus of the included articles from which the themes and categories were drawn from. The homogeneity of the themes and categories allowed the adoption of reciprocal translation and latterly the mapping of the various themes/categories in Phase 6. These themes/categories, which form the basis of what Noblit and Hare call “the line of argument”, are presented in the “RESULTS” section. The tabulated summaries are found in *Appendix* *6*.

### Stage 5: Analysing Data from Research and Non-research-based Sources

As the research team iteratively streamlined and organized the data, the expert team was critical in overseeing and guiding this process through numerous discussions. In so doing, the expert team considered that data from grey literature that was not quality-assessed or evidence-based could bias the discussion. As such, the research team thematically analyzed data from grey literature and non-research-based pieces such as letters, opinion and perspective pieces, commentaries and editorials extracted from the bibliographic databases. When these themes were compared with those from peer-reviewed data, no differences were identified.

### Stage 6: SSR Synthesis

The Best Evidence Medical Education (BEME) Collaboration guide ^49^ and the STORIES (Structured approach to the Reporting In healthcare education of Evidence Synthesis) statement ^61^ were adopted to guide the SSR narrative.

## RESULTS

A total of 10443 titles and abstracts were reviewed and a final 76 full-text articles included. Our thematic and directed content analyses yielded similar themes and categories. Following the Jigsaw Perspective and Funnelling stages, we categorized these themes as follows: characteristics of PIF in relation to professionalism (in medicine), role of socialization in PIF, enablers and barriers to PIF, and medical school approaches to supporting PIF.

1. PIF characteristics in relation to professionalism

PIF and professionalism are mutually reinforcing, each influencing the other. PIF is a necessary foundation to professionalism ^62^ while also contingent upon it. Professionalism in medicine is a process of adopting a shared belief system that focuses on improving the health of patients ^63,64^ by attaining technical and cognitive clinical competencies ^65–69^, meeting high ethical and moral standards ^64,67–71^, and displaying behaviours consistent with professional principles and values ^9,64,69,72–74^. To exemplify the profession’s expectations as a lifelong ideal, students must be able to reconcile their personal and professional identities.

Critical to the formation of professional identity, on the other hand, is a *commitment* to the profession ^62,72,75–77^. When this commitment deepens through an ongoing process of adapting, internalizing and assimilating professional traits into intrinsic characteristics (virtue-based professionalism) and observable actions (behaviour-based professionalism) ^2,5,6,68,69,76,78–80^, a new integrated identity takes shape ^2,5,6,68,69,76,78–80^. Factors that influence professional behaviour include mentorship and role-modeling ^81,82^, prevailing codes of conduct ^67^ and social and cultural concepts of the “good physician” ^82^. Manifesting such professional values and behaviours can further foster professional identity ^2,5,64,75–77,80,83^, through which medical students identify as a member of the profession ^67,84,85^ and aim to embody its roles and responsibilities ^2,6,8,9,62,63,65,66,68,69,72–75,80,83,85–96^.

Affirming the importance of professional attitudes, ethical conduct, reflective practice and supportive relationships, PIF thus captures the nuanced process by which a medical student personally and professionally transforms into a doctor ^2,97^.

2. Socialization in PIF

Socialization is the process of becoming a part of the medical community ^7,72,98^ and developing a sense of professional identity through shared knowledge and skills ^72,98^. This process is individualized, non-linear and heavily influenced by formal ^5,98,99^, informal and hidden curricula ^4,72,98^. As students move from early peripheral involvement ^8,72,100^ to assuming a more central role in the community of practice with increasing seniority ^69,72,98,101^, intrinsic characteristics, values, beliefs, behaviours and biases are re-examined ^62,63,69,80,93–96^, refined ^9,73,100^, re-aligned and integrated. Socialization is facilitated by formal ceremonies and seminal experiences such as White Coat Ceremony and cadaveric dissections ^62,69^, and promoted when experiencing patient care ^2,6,62,69,76,83,102^, managing clinical responsibilities ^8,72,100^, working long hours ^5^ and reflecting upon experiences and clinical identities ^5,69,71,83,96,99^. This evolving process, which continues along the continuum of medical education, sees individuals advance progressively from “doing” toward a way of “being” ^69^.

3. PIF influencing factors

A series of influencing factors promote or hinder professional identity formation as enablers or barriers that are intrinsic or extrinsic to the student. These are presented in Table 1 through the person-centric lens of RToP and its Individual, Relational and Societal Rings. Limited data on the Innate Ring prevented further evaluation of the impact of PIF on this aspect.

**Table 1:** Enablers and barriers to professional identity formation in medical school viewed through the RToP lens

	Intrinsic enablers	Extrinsic enablers	Barriers
	Medical student is able to:	Learning environment enables:	Student perceives or experiences:
Societal Ring	• Acknowledge societal expectations pertaining to professional role, responsibilities and codes of conduct ^10,67,72,73,101,105,118,119^ • Identify with medical professionals and wider healthcare community ^2,5,6,54,63–65,78,81,120,121^ • Exhibit professional behaviour in daily practice ^74^ • Fulfil entrusted responsibilities as a member of the healthcare team ^2,65,70,73,77,103,122^ • Build confidence with application of communication, counselling and clinical reasoning skills to contribute to the care of their patients ^73,103,122^	• Symbolic socialization events such as White Coat Ceremony or Honor Code ^62,67,69,118,123^ • Direct and repeat opportunities to interact with patients ^62,67,69,118,123^ • Meaningful professional relationships with multidisciplinary healthcare teams ^5,8–10,54,64,65,71,72,74,82,84,89,93,96,97,99,102–104,107,124^ • Clarity of role within team and wider healthcare system ^9^ • Formal curriculum to foster holistic, longitudinal knowledge acquisition and clinical education ^2,5–7,10,54,62–65,67,69,71–74,76–79,81–84,88,89,91,93–97,100–105,109,110,118–132^ • Art and humanities opportunities to foster creativity, acknowledge emotions and explore identities ^89,92,119^ • Hidden curriculum to align intended and enacted professional values and behaviours ^2,4–10,54,62,64,65,68–72,75,78–80,82–84,88–90,93–95,97,99,103–105,119,121,122,132,133^	• Disconnect between theoretical knowledge and application in clinical practice ^97,98^ • Heavy academic demands and competing responsibilities ^82,106,107,130^ • Tensions between personal values and broader professional identity instigated by challenging encounters ^4,7,65,81^ • Negative portrayal of profession by mainstream media or glamorization of traits such as cynicism ^68,99,124^ • Lack of opportunities or expectations to assume patient care responsibilities ^88,98,101,103^ • Difficulty navigating or fitting into new clinical environments ^7^
Relational Ring	• Develop professional relationships with patients, peers and team members ^72,84,88,97,124,132^	• Supportive clinical interactions between patients and students; students and doctors ^73,82,101,103,104,131,132^ • Collaborative relationships among peers ^74,81,84,101,128,129,131^ • Open and supportive discussions with faculty and peers ^65,73,74,81,129,131^ • Access to appropriate mentorship, advising and role modeling ^72,128^ • Guided reflective opportunities with feedback ^7,10^	• Mismatch between personality and values with those of team members or patients ^72,82,91,100^ • Challenging relationships with team member, patients or peers ^88^ • Hierarchical structures in clinical environment deterring students from seeking help or speaking up ^7,9,72,101^ • No mentors or role models ^72,128^
Individual Ring	• Show desire and sustain motivation to gain competence and engage in life-long learning ^88,101^ • Attend to emotions and engage in critical thinking and reflection ^2,4–6,9,10,54,62,64,66–68,71–74,78–81,84,86,91,92,94,99,102,105,107,109,110,121,123,125–128,134^	• Access to support systems including mentors and role models ^5,69,71,80,93,121^ • Exposure to challenging clinical experiences such as death and suffering ^118^ • Outlets for emotional and/or creative expression ^89,92,119^	• Tension between existing personal identity and aspiring professional identity ^7,65,81^ • Uncertainty or lack of confidence in clinical interactions that cast doubt on ability to fulfil professional tasks ^54,64,80,96,99^ • Unrealistic or conflicting expectations ^4,7,65,73,81,89^ • Absence of role models ^72,128^

**Table 2:** Strategies adopted by Medical Schools to support Professional Identity Formation

	Strategies adopted by medical schools to support PIF	References
Formal ethics and professionalism instruction	Prioritizing principles of professionalism and professional identity formation consistently through curricular goals (professional roles, codes of practice, patient-centred care, ethics instruction, cultural sensitivity, clinical reasoning, communication skills, interprofessional education) using relevant instructional methods (e.g. didactic classroom learning, online modules, seminars, lectures, tutorials, group projects, small-group discussions, reflective writing, experiential learning, community care), and including a system for timely and appropriate feedback to help students improve in clinical capabilities Performing formative and summative assessment of professionalism as opportunities for learning, remediation, and in extreme cases, exclusion if a student severely violates codes of conduct	^2,6,7,10,54,62,64,67,71,73,74,77,78,80,82,84,88,89,91,93–97,100,101,103–105,109,110,119–132,135^
Informal and hidden curriculum	Acknowledging the significant influence of informal interactions with the medical community, role models and patients during profound life moments such as birth, death or suffering on student learning, values, attitudes, behaviours, specialty choice or perceived suitability for medicine	^5,8–10,54,62,69,75,82–84,88–90,93,95,97,103–105,119,121,122,132,133^
Learning environment	Establishing guidelines to ensure safe and open learning environments in which learner confidentiality is maintained, student behaviours such as competing, comparing, interrupting, prescribing and speaking on behalf of another are mitigated; open and non-judgmental discourse supported; and professional behaviour reinforced as an indicator of future conduct	^81,84,91,107,123,132^
Symbolic socialization	Conducting contextually appropriate symbolic events such as White Coat Ceremony to foster socialization into the profession	^62,67,69,118,123^
Medical humanities	Formally incorporating humanities with modules as outlets for creative release and emotional expression through art and stories that support empathy, compassion, tolerance of uncertainty and critical thinking on issues such as ethics and social justice	^89,92,119^
Reflective practice	Enabling deliberate and guided reflection strategies using discourse and small-group discussions with feedback throughout students’ medical education to help them uncover assumptions, explore different perspectives, make sense of challenging encounters, grapple with ethical quandaries, manage difficult emotions or conflict, and construct and deconstruct values and identity through comparisons between lived experiences and prevailing narratives of meaning, all aiming to inform future actions and decisions	^2,5,6,9,10,54,62,64,66–68,71,73,74,78–81,84,86,91,92,94,99,102,105,107,109,110,121,123,125–128,134^
Stories and storytelling	Offering opportunities for students to recollect and verbalize stories of patient encounters, make meaning as events are recalled and structured (i.e. “storied”), shape a personal framework of caring, and develop a coherent physician ideal through critical reflection	^90,103,105,110,129^
Mentorship	Providing formal, purposeful, accessible, inclusive and longitudinal mentorship, as one-on-one or group mentoring models, with qualified faculty aware of power dynamics of interactions with students and equipped with appropriate mentoring skills including feedback to guide students reflect on experiences, navigate professional life, and assimilate knowledge into clinical practice	^3,54,66,80,81,88,93,99,102,104,124,129^
Role models	Cultivating positive role models (e.g. doctors, near-peers, residents, faculty, inter-professional team members) who support students’ psychological well-being, encourage reflection, support learning, and demonstrate decision-making and professional values and attitudes in clinical and non-clinical contexts	^5,8–10,54,64,71,72,74,80,82,84,89,93,96,97,99,102–104,107,118,124^
Non-medical influences	Acknowledging the role of family, prior experiences, medical dramas and societal perceptions on students’ personal values vs. professional expectations, and supporting students to mitigate dissonance and enhance alignment between professional development (e.g. professional attitudes, roles and behaviours) and internal bearings and identity (e.g. personal values), which if left unaddressed could lead to anxiety, frustration, and feelings of inadequacy	^72,84^

Intrinsic factors refer to the medical student’s attitudes, values, beliefs, moral and philosophical leanings and decision-making processes. Extrinsic factors relate to the clinical environment. Many factors influence how medical students reconcile their experiences ^7,9,67,72,103^ and reflections within the Individual Ring of ideals, values, beliefs, and personal and professional self-concepts ^4,91^, while interactions ^73,81,100,103–106^ impact Relational and Societal Rings. These rings are further affected by how experiences and reflections take shape in medical school. In the absence of effective, appropriate or adequate support ^7,72,98^, enabling factors such as reflection ^2,71,72,76,89,91,107^ or socialization ^69,72,98,101^ may become barriers that impede the merging of students’ personal and professional identities.

4. Medical School strategies to support PIF

Approaches that medical schools are taking to support PIF are presented in Table 2. The all-encompassing nature of these efforts signals an absence of clear or consistent approach across schools. What these reported strategies share is a foundation of pedagogical practices that view learning as a social construct, value role models, provide guided reflective practice, and institute longitudinal, inclusive and tailored forms of mentorship ^71,76,80^ within supportive learning environments ^7,9,67,72,103^ in which espoused and enacted values align. The formal ^5,98,99^ and hidden curricula ^4,72,98^ heavily influence students’ socialization into the medical community ^7,72,98^. As poignantly stated by Hodges et al.^108^, even if “a student can be prepared for excellent communication, collaboration, empathy, and patient-centered attitudes through years of formal training, just a few minutes in a work environment that does not model these behaviors will rapidly lead to their extinction in the student’s behaviors”. ^108^

## DISCUSSION

**Figure 3:**
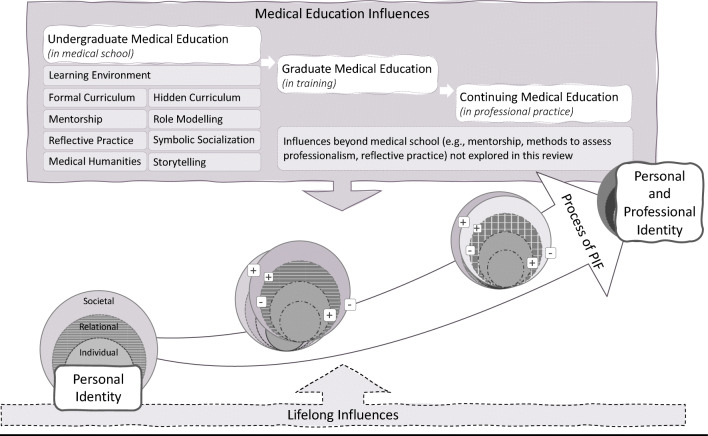
Integration of personal and professional identity entails a longitudinal, developmental process influenced by enabling (+) and disabling (−) factors that impact one’s personhood along the continuum of medical education

Findings from our review support the notion that PIF involves iterative construction, deconstruction and inculcation of professional beliefs, value systems and codes of conduct into a pre-existent concept of personhood. Students refine, reject or internalize new values, practices and behaviours while re-examining pre-existing ones. Such cycles of shaping and reshaping personal and professional identities are influenced by many factors including role models, reflections or responsibilities along the medical education continuum, as conceptualized in Figure 3. By viewing PIF through the RToP lens in this systematic scoping review, we identified a multitude of intrinsic and extrinsic factors that promote or impede individual, relational and societal aspects of a medical student’s personhood (Table 1). Importantly, if inadequately or inappropriately supported, enabling factors can become barriers to PIF.

As different aspects of a medical student’s personhood evolve in medical school, personal and professional beliefs or values may pose as competing forces. Making sense of complex or ambiguous experiences necessitates a critical ability to question assumptions, attend to emotions and explore different perspectives. Deconstructing the self to pursue congruence among multiple existing identities can be disorienting or disconcerting. Left to their own devices, learners may consider open questioning of assumptions socioculturally inappropriate, or find existing power relations unapproachable. They may arrive at incomplete or incorrect conclusions, experience feelings of inadequacy and impostorship, and withdraw from learning activities to avoid being “found out”. The complex process of PIF, as an outcome of medical education, is thus not a solitary or self-directed exercise for students to steer in a vacuum. While successful formation of a professional identity has been linked to career success, a mismatch between a person’s internal bearings and professional roles and expectations can create anxiety, frustration, and feelings of inadequacy, sometimes leading the individual to leave the profession ^109,110^.

To support PIF, medical schools are offering attention and action in multiple domains as encapsulated in Table 2. Any measure implemented by a medical school will by its nature affect students at societal and relational levels, with downstream effects that reshape individual and even intrinsic aspects of their personhood. Caring for the dying, for example, can influence medical students’ conceptions of life, death and religion. However, our review does not shed light on how everyday personal interactions ^111^, gender roles, online experiences ^72,84^, religious beliefs or existential philosophies shape students’ understanding of the profession and professional roles. Donning on a white coat does not sever a student’s personal proclivities, motivations and priorities. To do so would ignore the humanism and multifarious sources of influence upon a student’s life. There is a dearth of data on the influence of a student’s personal roles — as a child, spouse, parent, friend, member of the larger community — on their professional conduct and identity. Further literature on this angle could illuminate the extent to which experiences in one’s personal sphere may influence professional values, attitudes and behaviours.

Professionalism and PIF are bidirectionally related but distinct entities. At a time when unethical behaviour, burnout and suicide in clinical practice are on the rise ^31,112–115^, it is all the more essential for medical schools to explicitly promote their expectations and ideals of the profession through formal instruction, reflective opportunities, mentoring and feedback, aided by processes such as individualized developmental portfolios ^116,117^, along with a multi-faceted program of assessment. The challenge with the latter remains a lack of consistency and clarity on the constituent constructs within professionalism and PIF through established theoretical frameworks.

## LIMITATIONS

We acknowledge several limitations to this study. Guiding the analysis through the RToP lens is novel, and organization of factors within the four rings reflected the researchers’ own preconceptions. To reach consensus with minimal overlaps between and across categories required iterative communication to align our understanding of PIF influences and their relation to the rings. Further, despite a comprehensive search from snowballing of references and oversight from content experts, it is possible to have missed relevant literature. The included articles, by nature of scoping reviews, were of varying categories and caliber, and the majority represented Western perspectives, questioning generalizability within different contexts.

## CONCLUSION

PIF is a complex, non-linear and fluid process through which medical students navigate competing influences between their professional roles and personal lives, and iteratively construct and deconstruct evolving views of the self. In the absence of a unifying theoretical framework, we explored this process through the lens of personhood and encapsulated key factors that promote or hinder students’ identity development on individual, relational or societal levels. Also captured were the all-encompassing strategies that medical schools implement to support their students’ socialization into the profession. Deliberate efforts to foster inspiring mentored relationships and individualized guided reflections in supportive learning environments can foster the agency for students to harmonize their personal and professional identities over time, with the ultimate aim of improving practice on individual, institutional and societal levels.

## Supplementary Information

ESM 1 (DOCX 28 kb)

ESM 2 (DOCX 16 kb)

ESM 3 (DOCX 19 kb)

ESM 4 (DOCX 36 kb)

ESM 5 (PDF 13842 kb)

ESM 6 (PDF 427 kb)

## Data Availability

All data generated or analyzed during this review are included in this published article (and its appendices).

## **Abbreviations**

PIF: professional identity formation
RToP: ring theory of personhood
SEBA: systematic evidence-based approach
SSR: systematic scoping review
YLLSoM: Yong Loo Lin School of Medicine
NUS: National University of Singapore
NCCS: National Cancer Centre Singapore
PRISMA: Preferred Reporting Items for Systematic Reviews and Meta-Analyses
PICOS: population, intervention, comparison, outcome, study design
MERSQI: Medical Education Research Study Quality Instrument
COREQ: Consolidated Criteria for Reporting Qualitative Studies
BEME: Best Evidence Medical Education
STORIES: Structured approach to the Reporting In healthcare education of Evidence Synthesis
