# Supplementary material for: A Scoping Review of Professional Identity Formation in Undergraduate Medical Education
Source: J Gen Intern Med. 2021 Aug 16;36(11):3511–21. doi: 10.1007/s11606-021-07024-9 (PMC8606368; doi:10.1007/s11606-021-07024-9)
Supplement: Supplementary file 2 — (DOCX 16 kb) [file 11606_2021_7024_MOESM2_ESM.docx]

|  | | |
| --- | --- | --- |
| **PICOS** | **Inclusion Criteria** | **Exclusion Criteria** |
| Population | Undergraduate medical students  Postgraduate medical students | Residents and/or doctors within the clinical, medical, research and/or academic settings  Allied health specialties such as Pharmacy, Dietetics, Chiropractic, Midwifery, Podiatry, Speech Therapy, Occupational and Physiotherapy  Non-medical specialties such as Clinical and Translational Science, Alternative and Traditional Medicine, Veterinary, Dentistry |
| Intervention | Practices in supporting professional identity formation of medical students |  |
| Comparison | Comparisons of the various practices (approaches, modalities, processes, objectives, motivations, challenges, facilitating characteristics/resources) |  |
| Outcome | Approaches, modalities, processes, objectives, motivations, challenges, facilitating characteristics/resources in professional identity formation  Impact of supporting professional identity formation on students |  |
| Study design | Articles in English or translated to English  All study designs including:  -Mixed methods research, meta-analyses, systematic reviews, randomized controlled trials, cohort studies, case-control studies, cross-sectional studies, and descriptive papers  -Grey Literature / electronic and print information not controlled by commercial publishing  -Case reports and series, ideas, editorials, conference abstracts, and perspectives  Year of Publication: 1 January 2000 – 1 July 2020  Databases: PubMed, Embase, PsycINFO, ERIC, Scopus | Articles focusing on non-human subjects |
