# Supplementary material for: A Scoping Review of Professional Identity Formation in Undergraduate Medical Education
Source: J Gen Intern Med. 2021 Aug 16;36(11):3511–21. doi: 10.1007/s11606-021-07024-9 (PMC8606368; doi:10.1007/s11606-021-07024-9)
Supplement: Supplementary file 3 — (DOCX 19 kb) [file 11606_2021_7024_MOESM3_ESM.docx]

**SCOPUS 176**

( TITLE-ABS-KEY ( medicine OR medical OR clinical OR residen* OR physician* ) AND TITLE-ABS-KEY ( professional AND identity OR professional AND identities OR identity AND formation OR identity AND formations ) AND TITLE-ABS-KEY ( teaching OR teachings OR curriculum OR curricula OR pedagog* ) )

**EMBASE 2323**

('medical education'/exp OR 'clinical education'/exp) AND ('curriculum'/exp OR curricula:ti,ab OR curriculum:ti,ab OR teaching:ti,ab OR teachings:ti,ab OR pedagog*:ti,ab) AND ('professional identity'/exp OR 'professional standard'/exp OR 'professional identity':ti OR 'professional identities':ti OR 'identity formation':ti OR 'identity formations':ti)

**PUBMED 4063**

(("Professionalism"[Mesh] OR "Social Identification"[Mesh] OR “Professional Role”[MeSH] OR "Social Values"[Mesh] OR professional identit*[tiab] OR professionalism*[tiab] OR e-professionalism[tiab] OR identity formation[tiab] OR socialization[tiab])) AND ("Education, Medical"[Mesh] OR ((“Teaching/methods"[Mesh] OR "Curriculum"[Mesh] OR curricula[tiab] OR curriculum[tiab] OR teaching[tiab] OR teachings[tiab] OR pedagog*[tiab]) AND (medical[tiab] OR medicine[tiab] OR clinical[tiab] OR residen*[tiab] OR physician*[tiab])))

**ERIC 149**

“medical education” AND “professional identity”

**PSYCINFO 1155**

#1

exp Medical Education/ or 'medical education'.ti,ab.

#2

(exp Curricula/ or curricula.ti,ab. or curriculum.ti,ab. or teaching.ti,ab. or teachings.ti,ab. or teach.ti,ab. or teacher.ti,ab. or teachers.ti,ab. or tutor.ti,ab. or tutors.ti,ab. or tutoring.ti,ab.) AND (Physician*.ti,ab. or resident.ti,ab. or residen*.ti,ab or doctor.ti,ab. or doctors.ti,ab or medical.ti,ab. or medicine.ti,ab.)

#3

(exp identity formation/ OR exp social identity/) or ('professional identity'.ti,ab. OR 'professional identities'.ti,ab.OR 'identity formation'.ti,ab. OR 'identity formations'.ti,ab.OR professionalism.ti,ab.)

**(1 OR 2) AND 3**
