# Supplementary material for: A Scoping Review of Professional Identity Formation in Undergraduate Medical Education
Source: J Gen Intern Med. 2021 Aug 16;36(11):3511–21. doi: 10.1007/s11606-021-07024-9 (PMC8606368; doi:10.1007/s11606-021-07024-9)
Supplement: Supplementary file 4 — (DOCX 36 kb) [file 11606_2021_7024_MOESM4_ESM.docx]

106 full-text articles

Database search:

- PubMed 6724
- Embase 2247
- PsycINFO 1155
- ERIC 149
- Scopus 168

Total: 10443 articles

9747 full text articles

Excluded 696 duplicate articles

272 full-text articles

Excluded non-relevant articles based on title and abstract (e.g. Professional Identity Formation not a major topic)

Excluded articles based on exclusion criteria

- Allied health specialties such as Pharmacy, Dietetics, Chiropractic, Midwifery, Podiatry, Speech Therapy, Occupational and Physiotherapy
- Non-medical specialties such as Clinical and Translational Science, Alternative and Traditional Medicine, Veterinary, Dentistry
- Articles focusing on non-human subjects

Excluded articles based on exclusion criteria

- Residents and/or doctors within the clinical, medical, research and/or academic settings

76 full-text articles
