# Supplementary material for: A Scoping Review of Professional Identity Formation in Undergraduate Medical Education
Source: J Gen Intern Med. 2021 Aug 16;36(11):3511–21. doi: 10.1007/s11606-021-07024-9 (PMC8606368; doi:10.1007/s11606-021-07024-9)
Supplement: Supplementary file 6 — (PDF 427 kb) [file 11606_2021_7024_MOESM6_ESM.pdf]

Appendix 6. *Tabulated Summaries*

S/N	Author(s)	Title	Year	Overview/Aim	Article Type/ Methods	Main Empirical Findings	Insights Drawn
**1**	Aagaard & Moscoso	Practical implications of compassionate off- ramps for medical students	2019	*"Attrition from medical school remains uncommon even when a medical student performs poorly, has a change in interests, or experiences an unexpected life event that alters his/ her ability to succeed as a physician. In this commentary, [the authors] describe how a representative student may benefit from Bellini and colleagues' recommendations."*	Commentary	*NIL*	*"[The authors] believe Bellini and colleagues' recommendations will help schools achieve the moral imperatives of humanistic care for students while honoring the social contract of the medical profession."*<br><br>*"Recommendations include:<br>(1) Implement Competency-Based Education and Training to Identify Struggling Learners<br>(2) Enable Ongoing Assessment of Commitment to Career Path via a Professional Identity Formation Curriculum<br>(3) Use Career Advisors and Coaches Who Understand Alternative Career Pathways<br>(4) Provide Credit or Credential for Competencies Already Achieved (e.g., Certificates, Master's Degrees<br>(5) Require Financial Counseling and Support Debt Forgiveness<br>(6) Require Medical Schools to Report on Their Remediation Programs and Handling of Debt"*
**2**	Ahmad et al.	Nurturing professional identity through a community based education program: medical students experience	2018	*"Community-based education (CBE) has an impact on the types of medical students produced at the end of medical training. However, its impact on professional identity development has not been clearly understood. This study thus explores the effect of the CBE program on PI through a study conducted on a group of Universiti Sains Malaysia medical students who had finished the Community and Family Case Study (CFCS) program."*	Qualitative phenomenological study; focus group discussions and student reflective journals	*"Personal, role, social, and research identities generated contribute to the professional identity development of medical students. CFCS program nurtured personal identity through the development of professional skills, soft skills, and personal values; role identity in terms of primary care of patients and interprofessional awareness; social identity in terms of community awareness related to culture, society, and politics; and research identity in terms of research skills in epidemiology and research methodology."*	*"A community-based education program can help to promote professional identity development amongst medical students."*
**3**	Azmand et al.	Learning professionalism through hidden curriculum: Iranian medical students' perspective	2018	*"Learning professionalism is a central topic in medical education. While many factors could affect the educational process of professionalism, the hidden curriculum is considered one of the most important ones. As the working components of a hidden curriculum might be specific to the settings, this study explored its components in terms*	Qualitative study; semi-structured and in-depth interviews	*"Seven main themes were extracted as the working components of a hidden curriculum regarding professionalism: 'convenient patients', 'evaluate me', 'trust as the base of team interactions', 'perceiving encouragement', 'relationship satisfaction and authenticity', 'workload and students'*	*"Students' perception and experiences are a rich source of gaining a deeper understanding of the working hidden curriculum. In this study, two groups of human-related and environment-related elements were extracted. They were effective in the formation of the current 'ethical climate', which shaped the professional and ethical identity of medical*

Appendix 6. <u>*Tabulated Summaries*</u>

				of professionalism and ethical conduct from the viewpoint of Iranian undergraduate medical trainees."		*well-being' and 'role modelling at the heart of professionalism'."*	*trainees. Moreover, specific plans regarding the condition of the settings may provide opportunities for medical educators to enhance professionalism in their institutions."*
**4**	Barone et al.	Supporting the development of professional identity in the millennial learner	2019	*"An awareness of professional identity development helps teachers understand some of the workplace differences noted between generations, particularly with millennial learners. Continuing the Council on Medical Student Education in Pediatrics series on great clinical teachers, [the authors] focus on supporting professional identity formation in millennial learners. This paper outlines case studies with the use of the I-CA^{2}R^{2}E model (individual connection, create, acknowledge and adjust, reflect and role model, and exchange) to provide strategies to maximize learners' professional identity formation. Two case studies are presented."*	Commentary	*NIL*	*"The early stages of a medical learner's professional identity formation can be disconcerting and filled with conflict, self-doubt, and an inadequate sense of belonging to the profession. Millennial learners may particularly need guidance through challenges to make critical decisions (ordering and reordering priorities) that ultimately lead to the embodiment of professional behaviors (ie, thinking, acting, and feeling like a physician). Supervisors who approach teachable moments with open-mindedness, a willingness to challenge expectations, and tools to stimulate exploration and self-reflection (I-CA^{2}R^{2}E) will be able to promote learners' development of a strong foundational identity in medicine."*
**5**	Barr et al.	Developing a patient focussed professional identity: An exploratory investigation of medical students' encounters with patient partnership in learning	2015	*"Patient encounters are central to the provision of learning opportunities for medical students and their development as medical professionals. The primary aim of the study reported in this paper was to discover how partnering medical students with patients with chronic illness in undergraduate learning influenced the development of a patient centred professional identity and professionalism. The study was conducted with Year 5 and 6 students at a medical clinical school in Australia."*	Qualitative study; focus groups, extended response questionnaire and semi-structured interviews	*"The professional identity of medical students is constructed along traditional lines in the preclinical years. Patient-partnership offers a disruption to this development by way of an intersection with patients with chronic illness which potentially allows meaningful construction of what a patient-centred identity should be. This point of reflection provides an opportunity to engage at a higher level in medical identity development and professionalism"*	*"The findings discussed in this paper further stimulate the patient-centred agenda by understanding the conflict associated with the student–patient nexus in medical education and its potential for building professionalism and a patient-centred professional identity. To continue the drive for a patient-centred professional identity there must be ongoing engagement with patients in medical education, preferably commencing early in a student's journey so that it becomes the expected norm. This study has highlighted that a true patient-centred emphasis is being encountered too late in their socialization process."*
**6**	Beck et al.	Professional identity development through service learning: a qualitative study of first-year medical students volunteering at a medical specialty camp	2015	*"Despite the proliferation of speciality camps in medical education where medical students volunteer in a camp setting for children with a variety of medical conditions, there is lack of systematic assessment. [The authors] studied the experiences and perspectives of medical students volunteering at a specialty camp and the potential impact it had on them and their professional identity formation."*	Qualitative study; focus groups and follow-up individual interviews	*"Students described their experience as motivating and career reinforcing. It helped them "move beyond the textbook" and deepened their commitment to serving future patients with compassion."*	*"Medical schools could use camps as a promising community service-learning experiences to foster professional identity formation."*

Appendix 6. *Tabulated Summaries*

**7**	Bogetz & Bogetz.	An evolving identity: how chronic care is transforming what it means to be a physician	2015	*"Physician identity and the professional role physicians play in health care is rapidly evolving. Over 130 million adults and children in the USA have complex and chronic diseases, each of which is shaped by aspects of the patient's social, psychological, and economic status. To date, physician professional identity formation has centered on autonomy, authority, and the ability to "heal." These notions of identity may be counterproductive in chronic disease care, which demands interdependency between physicians, their patients, and teams of multidisciplinary health care providers."*	Commentary	*NIL*	*"[The authors] believe that early training on the physician's role, the strategic use of professional power, and the effective utilization of patients as members of the health care team should be a required component of all medical training. This education should be introduced early and be explicitly reinforced over time as trainees enter new stages of their professional development. Medical educators must develop and implement pedagogical models that emphasize shared responsibility, collaboration, and systems-based practice. Additionally, educators must cultivate in trainees a willingness to act as humble partners with patients through concrete clinical experiences that broaden thinking about diseases and the physical, social, and psychological impact they have on their patients' lives."*
**8**	Boudreau & Fuks	The humanities in medical education: ways of knowing, doing and being	2014	*"The personhood of the physician is a crucial element in accomplishing the goals of medicine. [The authors] review claims made on behalf of the humanities in guiding professional identity formation and explores the dichotomy that has evolved, since the Renaissance, between the humanities and the natural sciences."*	Perspective paper	*NIL*	*"The result of this dichotomy is an historic misconstrual, preoccupying educators and diverting them from the moral development of physicians. [The authors] propose a curricular framework based on the recovery of Aristotelian concepts that bridge identity and activity. The humanities and the natural sciences, jointly and severally, can fulfil developmental, characterological and instrumental purposes*
**9**	Boudreau et al.	Physicianship: educating for professionalism in the post-Flexnarian era	2011	*""Physicianship" represents a unified approach to the education of future practitioners based upon a core set of definitions of professionalism and healing and a list of desired attributes that are seen to permeate the entire educational experience—from the admission of medical students, to their teaching and learning activities, and ultimately to the assessment of behaviors in teachers and learners. Although he did not write extensively about professionalism, Abraham Flexner clearly understood its critical role in medical practice. [The authors] opine that a curriculum based on "Physicianship"- the physician as healer and professional - can serve as a logical post-Flexnerian curriculum."*	Perspective paper	*NIL*	*"The conceptual armature of Physicianship and the attributes necessary for the fulfilment of both the professional and healer role can assist in the selection of students and constitute the educational blueprint for medical teaching. The critically important concepts of identity formation and the requirements for the valid and reliable assessment of professional behaviors of students and faculty are essential components. [The authors] suggest that a Physicianship curriculum, as conceived and deployed at the McGill University Faculty of Medicine, might resonate with Flexner."*

Appendix 6. *Tabulated Summaries*

**10**	Boudreau et al.	Affirming professional identities through an apprenticeship: insights from a four-year longitudinal case study	2014	*"A four-year course, entitled Physician Apprenticeship, was introduced at McGill University's Faculty of Medicine in 2005. The primary objective of the course is to assist students in their transition from laymen to physicians."*	Mixed methods study	*"Physician Apprenticeship activities promoted and sustained medical professionalization in the participants. Salient features of successful apprenticeship learning were access to authentic clinical experiences as well as the provision of a safe learning environment and guided critical reflection. The latter two ingredients appear to be mutually reinforcing and contributed to the creation of meaningful student-teacher relationships. Teachers exhibited several qualities that align with a parental role. Students became increasingly aware of having entered the kinship of physicians. Teachers experienced a renewal and validation of their commitment to the ideals of medicine."*	*"Findings strongly suggest that a longitudinal apprenticeship in an undergraduate medical program can contribute to the formation and reaffirmation of professional identity. Dedicated fellows are required to reproduce this result."*
**11**	Byars et al.	A curricular addition using art to enhance reflection on professional values	2015	*"Art and humanities can enhance undergraduate medical education curricular objectives. Most commonly, art is used to help students learn observational skills, such as medical interviewing and physical diagnosis. Educators concurrently struggle to find ways to meaningfully teach professional values within crowded curricula. [The authors] proposed a curriculum aimed to combine art and reflection to actively convey tenets of medical professionalism."*	Descriptive paper	*NIL*	*"The use of artwork as a vehicle to enhance reflection on professional values has provided the clerkship with a way to explicitly teach professional values while maximizing student engagement through utilization of their unique individual experiences. It is grounded in educational theory and has been incorporated into the existing clerkship structure with preliminary data suggesting good acceptance by students and faculty." "Artwork can enhance student reflection on professional values by engaging the educational constructs of conscious observation, emotional engagement, reflective practice, and group discussion."*
**12**	Bynum & Artino	Who am I, and who do I strive to be? Applying a theory of self-conscious emotions to medical education	2018	*"The self-conscious emotions of shame, guilt, and pride are a distinct set of cognitively complex, powerful, and ubiquitous emotions that arise when an individual engages in self-evaluation. Currently, little is known about the influence or outcomes of self-conscious emotions in medical learners. In this article, [the authors] present a leading theory of self-conscious emotions that outlines the appraisals and attributions that give rise to and differentiate shame, guilt, and two forms of pride."*	Perspective paper	*NIL*	*"[The authors] apply the theory to three relevant topics in medical education: perfectionism, professional identity formation, and motivation. In doing so, [the authors] present novel ways of viewing these topics through the lens of self-conscious emotion, suggest areas of future research, and outline a framework for emotional resilience training. Ultimately, the goal of this article is to highlight the fundamental nature of shame, guilt, and pride, which [the authors] believe are underappreciated and understudied in medical education, and to inform future*

Appendix 6. *Tabulated Summaries*

							empirical study on the role that these emotions might play in medical education. Additionally, from a practical standpoint, this article aims to encourage educators and learners to recognize self-conscious emotions in themselves and their colleagues, and to begin developing more resilient approaches to learning-approaches that acknowledge and confront shame, guilt, and pride in medical education."
**13**	Byszewski et al.	Socialization to professionalism in medical schools: a Canadian experience	2015	*"Accrediting bodies now recognize the importance of developing the professionalism competency, by setting standards that require medical schools to identify where professionalism is addressed and how it is evaluated within the formal curriculum. The objective of this study was to compare how professionalism competency is formally addressed in the curricula of Canadian medical schools, and to better understand the Canadian approach to reporting and remediation of lapses."*	Qualitative study; questionnaires, in-depth telephone interviews and focus group.	*"The preponderance of formal professionalism teaching remains in the form of lectures and small group sessions in the preclinical years. Formal teaching declines significantly in the clerkship/clinical years. Evaluation is usually performed by a clinical supervisor, but OSCE, portfolio, and concern notes are increasingly used. Role modeling is heavily relied upon in clinical years, suggesting faculty training can help ensure clinical teachers recognize their influence on trainees. Formal remediation strategies are in place at most schools, and often involve essay writing, reflection exercises, or completion of learning modules about professionalism. Lack of clarity on what defines a lapse and fear of reprisal (for both trainees and faculty) limits reporting."*	*"This study provides an overview of how professional identity formation is supported in the Canadian context, guided by the standards set out by CanMEDS. Despite a rich literature that describes the definition, program design and evaluation methods for professionalism, in some areas of the curriculum there is still an opportunity to ensure programs embrace the suggested framework. Examples of teaching and evaluation methods, deficiencies in the clinical years of study (clerkship) and challenges in addressing lapses and organizational structure are identified. The results help identify the gaps that need to be addressed and some solutions that can be modeled at other academic institutions."*
**14**	Cave & Clandinin	Revisiting the journal club	2007	*"Recent descriptions of journal clubs identify their purpose as reading current medical literature, critically appraising it for validity and applicability to the readers' patient population, and distilling the best available clinical evidence. A clinical problem or question from practice within a discipline is identified, and relevant literature is selected and critically appraised. The process addresses the first tenet of evidence-based medicine; that is, gathering the best evidence from research data, but there is little information about when and how the second and third tenets (namely, incorporating individual clinician's*	Qualitative study	*"Journal clubs facilitated learning around two neglected tenets of evidence-based medicine: the integration of clinical expertise, and incorporating patients' perspectives into clinical decision-making. It also fulfilled an earlier purpose of journal clubs, namely the fostering of collegiality and the development of professional identity in physicians."*	*"This study shows the value of reading medical literature that is different, but complementary, to the kind read in contemporary journal clubs."*

Appendix 6. *Tabulated Summaries*

				expertise and individual patient's perspective) are addressed."			
**15**	Chuang et al.	To the point: reviews in medical education—taking control of the hidden curriculum	2010	*"The hidden curriculum is poorly defined but it plays an integral role in professional identity formation."*	Perspective paper	*NIL*	*"The hidden curriculum should be shaped and not eliminated. It can serve as a tool to teach students professional values. The hidden curriculum has the potential to bring about a lot of negatives if left unchecked. However the success of these methods of regulating the hidden curriculum depends on the learner too, on whether they want to/are able to self-reflect appropriately."*
**16**	Crues et al.	Supporting the development of a professional identity: general principles	2019	*"While teaching medical professionalism has been an important aspect of medical education over the past two decades, the recent emergence of professional identity formation as an important concept has led to a re-examination of how best to ensure that medical graduates come to "think, act, and feel like a physician." If the recommendation that professional identity formation as an educational objective becomes a reality, curricular change to support this objective is required and the principles that guided programs designed to teach professionalism must be re-examined."*	Descriptive paper	*NIL*	*"It is proposed that the social learning theory communities of practice serve as the theoretical basis of curricular changes that support professional identity formation include. These include: the necessity to establish identity formation as an educational objective, include a cognitive base on the subject in the formal curriculum, to engage students in the development of their own identities, provide a welcoming community that facilitates their entry, and offer faculty development to ensure that all understand the educational objective and the means chosen to achieve it. Finally, there is a need to assist students as they chart progress towards becoming a professional."*
**17**	Cruess & Cruess	Teaching professionalism – Why, What and How	2011	*"Due to changes in the delivery of health care and in society, medicine became aware of serious threats to its professionalism. Beginning in the mid-1990s it was agreed that if professionalism was to survive, an important step would be to teach it explicitly to students, residents, and practicing physicians. This has become a requirement for medical schools and training programs in many countries."*	Perspective paper	*NIL*	*"Teaching of professionalism must be both explicit and implicit. The cognitive base consisting of definitions and attributes and medicine's social contract with society must be taught and evaluated explicitly. Of even more importance, there must be an emphasis on experiential learning and reflection on personal experience. The general principles, which can be helpful to an institution or program of teaching professionalism, are presented, along with the experience of McGill University, an institution which has established a comprehensive program on the teaching of professionalism."*
**18**	Cruess et al.	Amending Miller's Pyramid to include professional identity formation	2016	*"In 1990, George Miller published an article entitled "The Assessment of Clinical Skills/ Competence/ Performance" that had an immediate and lasting impact on medical education. In his classic article, he*	Commentary	*NIL*	*"The recent emphasis on professional identity formation has raised questions about the appropriateness of "Does" as the highest level of aspiration. It is believed that a more reliable indicator of professional behavior is the incorporation of the values*

				stated that no single method of assessment could encompass the intricacies and complexities of medical practice. To provide a structured approach to the assessment of medical competence, he proposed a pyramidal structure with four levels, each of which required specific methods of assessment. As is well known, the layers are "Knows," "Knows How," "Shows How," and "Does." Miller's pyramid has guided assessment since its introduction; it has also been used to assist in the assessment of professionalism."			*and attitudes of the professional into the identity of the aspiring physician. It is therefore proposed that a fifth level be added at the apex of the pyramid. This level, representing the presence of a professional identity, should be "Is," and methods of assessing progress toward a professional identity and the nature of the identity in formation should be guided by currently available methods."*
**19**	Cruess et al.	Reframing medical education to support professional identity formation	2014	*"Teaching medical professionalism is a fundamental component of medical education. The objective is to ensure that students understand the nature of professionalism and its obligations and internalize the value system of the medical profession. The recent emergence of interest in the medical literature on professional identity formation gives reason to re-examine this objective. The unstated aim of teaching professionalism has been to ensure the development of practitioners who possess a professional identity. The teaching of medical professionalism therefore represents a means to an end. The principles of identity formation that have been articulated in educational psychology and other fields have recently been used to examine the process through which physicians acquire their professional identities."*	Perspective paper	*NIL*	*"[The authors] recommend that, as Merton postulated many years ago, the principal objectives of medical education should be to ensure that each practitioner has acquired both the knowledge and skills necessary for the practice of medicine and a professional identity so that he or she comes to think, act, and feel like a physician. Within this conceptual framework, teaching professionalism becomes not an end in itself but a means to an end. [The authors] believe that the end has always been the development of a professional identity and that the substance of that identity should be the "good physician." In reframing the educational goal to support and assist learners as they develop their professional identities, the emphasis shifts to an interpretation of professionalism based on 'being' rather than 'doing'."*
**20**	Cruess et al.	A schematic representation of the professional identity formation and socialization of medical students and residents: A guide for medical educators	2015	*"Recent calls to focus on identity formation in medicine propose that educators establish as a goal of medical education the support and guidance of students and residents as they develop their professional identity. Those entering medical school arrive with a personal identity formed since birth. As they proceed through the educational continuum, they successively develop the identity of a medical student, a resident, and a physician. Each*	Perspective paper	*"Each learner reacts to different factors in her or his own fashion, with the anticipated outcome being the emergence of a professional identity. However, the inherent logic in the related processes of professional identity formation and socialization may be obscured by their complexity and the large number of factors involved."*	*"Drawing on the identity formation and socialization literature, as well as experience gained in teaching professionalism, [The authors] developed schematic representations of these processes. They adapted them to the medical context to guide educators as they initiate educational interventions, which aim to explicitly support professional identity formation and the ultimate goal of medical education—to ensure that medical students*

Appendix 6. <u>*Tabulated Summaries*</u>

				individual's journey from layperson to skilled professional is unique and is affected by "who they are" at the beginning and "who they wish to become".'			*and residents come to 'think, act, and feel like a physician',"*
**21**	Day et al.	What experiences in medical school trigger professional identity development?	2019	*"This qualitative inquiry used conceptual change theory as a theoretical lens to illuminate experiences in medical school that trigger professional identity formation. According to conceptual change theory, changes in personal conceptualizations are initiated when cognitive disequilibrium is introduced. [The authors] sought to identify the experiences that trigger cognitive disequilibrium and to subsequently describe students' perceptions of self-in-profession prior to the experience; the nature of the experience; and, when applicable, the outcomes of the experience."*	Qualitative study; focus groups, individual interviews, and students' comments from course and end-of-year evaluations	*"[The authors] identified four experiences that triggered cognitive disequilibrium in relationship to students' perceptions of self-in-profession: (a) transition from undergraduate student to medical student, (b) clinical experiences in the preclinical years, (c) exposure to the business of medicine, and (d) exposure to physicians in clinical practice."*	*"[The authors] believe these experiences represent vulnerable periods of professional identity formation during medical school. Educators interested in purposefully shaping curriculum to encourage adaptive professional identity development during medical school may find it useful to integrate educational interventions that assist students with navigating the disequilibrium that is introduced during these periods."*
**22**	Easton et al.	How medical teachers use narratives in lectures: a qualitative study	2016	*"There are strong theoretical arguments for using narratives in teaching and learning within medicine, but little is known about how they are used in medical lectures."*	Qualitative study: observation of three medical lectures, individual interviews with lecturers, and separate focus group interviews with medical students.	*"Lecturers used a variety of narratives on a range of themes, from clinical cases to patient experience narratives or narratives about their professional careers. The findings support existing literature which suggests that narratives may be a useful tool for learning in medicine. This study suggests that narratives tap into several key learning processes including providing a relevant context for understanding, engaging learners, and promoting memory."*	*"For medical students in lectures, narratives may be particularly relevant in promoting humanistic aspects of medicine, including professional identity and empathy."*
**23**	Findyartini et al.	Remediating lapses in professionalism among undergraduate pre-clinical medical students in an Asian Institution: a multimodal approach	2018	*"Fostering personal identity formation and professional development among undergraduate medical students is challenging. Based on situated learning, experiential learning and role-modelling frameworks, a six-week course was developed to remediate lapses in professionalism among undergraduate medical students. This study aims to explore the students' perceptions of their personal identity formation and professional development following completion of the course. In the pilot course, field work, role-model*	Qualitative phenomenological study; reflective diaries	*"Three main themes were revealed. First, students highlighted the strength of small group activities in helping them 'internalise the essential concepts'. Second, the role-model shadowing supported their understanding of 'what kind of medical doctors they would become'. Third, the field work allowed them to identify 'what the "noble values" are and how to implement them in daily practice'."*	*"By implementing multimodal activities, the course has high potential in supporting personal identity formation and professional development among undergraduate pre-clinical medical students, as well as remediating their lapses in professionalism. However, there are challenges in implementing the model among a larger student population and in documenting the long-term impact of the course."*

				shadowing and discussions with resource personnel were conducted."			
**24**	Frost et al.	I am a doctor: negotiating the discourses of standardization and diversity in professional identity construction	2013	*"Medical educators have expressed concern that students' professional identities do not always align with their expectations or with professional standards. [The authors] propose that, in constructing appropriate professional identities, medical students today are affected by the competing discourses of diversity and standardization."*	Review	*"The discourse of diversity emphasizes individuality, difference, and a plurality of possibilities and advances the notion that heterogeneity is beneficial to medical education and to patients. In contrast, the discourse of standardization strives for homogeneity, sameness, and a limited range of possibilities and conveys that there is a single way to be a competent, professional physician. Thus, these discourses are in tension, a fact that medical educators largely have ignored. A social constructionist approach to identity suggests that medical students resolve this tension in different ways and construct different identities as a result."*	*"To influence medical students' professional identity construction, [the authors] advocate that educators seek change across the profession—faculty must acknowledge and take advantage of the tension between the discourses of standardization and diversity."*
**25**	Gaufberg et al.	In pursuit of educational integrity: professional identity formation in the Harvard Medical School Cambridge Integrated Clerkship	2017	*"Graduates of Harvard Medical School's Cambridge Integrated Clerkship (CIC) describe several core processes that may underlie professional identity formation: encouragement to integrate pre-professional and professional identities; support for learner autonomy in discovering meaningful roles and responsibilities; learning through caring relationships; and a curriculum and an institutional culture that make values explicit."*	Qualitative study; semi-structured interviews	*"[The authors] suggest that the benefits of educational integrity accrue when idealistic learners inhabit an educational model that aligns with their own core values, and when professional development occurs in the context of an institutional home that upholds these values."*	*"Medical educators should clarify and animate principles within curricula and learning environments explicitly in order to support the professional identity formation of their learners."*
**26**	Goldie	The formation of professional identity in medical students: considerations for educators	2012	*"Medical education is about more than acquiring an appropriate level of knowledge and developing relevant skills. To practice medicine, students need to develop a professional identity – ways of being and relating in professional contexts."*	Perspective paper	*NIL*	*"Identity is multiple, dynamic, relational, situated, embedded in relations of power, yet negotiable. Students' professional identity formation is influenced more by the informal and hidden curricula than formal teaching experiences. Interaction with appropriate role models; opportunities to experiment and receive feedback on provisional identities and be provided with the pedagogical space to understand and synergise developing identities, is recommended. The profession must be proactive in identifying suitable role models and integrating students into the various social networks existing within medical schools. Helping students form, and successfully integrate their professional*

Appendix 6. <u>*Tabulated Summaries*</u>

							selves into their multiple identities, is a fundamental of medical education."
**27**	Gormley et al.	What ... you can't tell left from right?' Medical students' experiences in making laterality decisions	2019	*"Wrong-sided procedures represent some of the most catastrophic errors in health care. Although errors in laterality are multifaceted in origin, human error is considered to be an important root cause. Evidence suggests that a significant proportion of the population, including medical students, experience difficulty in left–right discrimination (LRD). Given that not all medical students have equal LRD ability, there have been calls to raise awareness of this issue in medical education. The experiences of medical students with LRD, including those who have difficulty, remain unknown. A qualitative study was conducted using hermeneutic phenomenology. Medical students with a wide range of abilities in LRD were invited to participate and to be interviewed."*	Qualitative study; interviews	*"Analysis yielded four main themes: (i) discriminating right from left: an unconscious or a conscious task? (ii) 'What . . . you can't tell right from left?': an undesirable skill deficit; (iii) concealment, and (iv) 'But you're going to be a doctor!': impact on professional identity formation."*	*"This study challenges normative expectations that LRD is an effortless task for all. Individuals who are challenged in LRD must engage in a complex conscious process to determine right from left. For the most part, this process is relatively effortless. However, the context of being a medical student can impose extra demands and heightens the risk associated with potential error. Medical education needs to respond by raising the profile of this challenge, with which many of the medical students are confronted, and by extending support to assist them in the interests of safe patient care."*
**28**	Grasset et al.	Medical students' professional identity development from being actors in an objective structured teaching exercise	2018	*"Medical students develop professional identity through structured activities and impromptu interactions in various settings. [The authors] explored if contributing to an Objective Structured Teaching Exercise (OSTE) influenced students' professional identity development."*	Qualitative study; focus group interviews	*"On an institutional level, students developed a feeling of belonging to the institution. At an interactional level, students realized they could influence the teaching interaction by actively seeking or giving feedback. On the personal level, students realized that errors could become sources of learning and felt better prepared to receive faculty feedback."*	*"Taking part in OSTEs as a simulated resident has a positive impact on students' vision regarding the institution as a learning environment and their own role by actively seeking or giving feedback. OSTEs support their professional identity development regarding learning and teaching while sustaining faculty development."*
**29**	Hafferty et al.	Alternative framings, countervailing visions: locating the "p" in professional identity formation	2016	*"Professional identity formation in medical education is referenced increasingly as an object for educational reform. [The authors] introduce core concepts from two largely untapped literatures on identity and formation, contrasting framings on occupational preparation from within the organizational socialization literature with issues of socialization and professional acculturation from a military sciences perspective."*	Commentary	*NIL*	*"The organizational sciences literature emphasizes socializing a workforce to "fit in," raising questions about how organization values might clash with core professional values concerning patient primary and social justice. The military literature, in turn, advances the notions of professional identity as a collective property, and that a particular social other (the public) must participate in shaping the group's identity as a profession. [The authors] extrapolate from these reviews that the training of physicians- as-professionals, and thus issues of socialization and identity formation, require intentionality and specificity around these contrasting issues.*

Appendix 6. <u>*Tabulated Summaries*</u>

							In turn, they argue that medical educators must attend to socializing trainees to a professional group identity while at the same time producing health care professionals who retain the capacity to resist the bureaucratic application of standardized solutions to contemporary problems."
**30**	Hargreaves	Reflection in medical education	2016	*"This paper encapsulates the strands of reflection in learning and "becoming" a reflective practitioner, which may apply to many disciplines and professions, but is specifically contextualised here to medical education and an effective career in healthcare. The paper focuses on the reasons for and examples of facilitating reflective practice, and considers future directions for reflection."*	Perspective paper	*NIL*	*"The development of reflective practice is certainly crucial for formative learning at the undergraduate stage of medical education, and throughout the clinician's continuing professional development. Tutors and students would benefit from open discussion of the metacognitive dimensions of learning and the theories underpinning the process of reflection to facilitate their reflective competence."*
**31**	Hatem et al.	Becoming doctors: examining student narratives to understand the process of professional identity formation within a learning community	2019	*"Professional identity formation is a key aim of medical education, yet empiric data on how this forms are limited. This study is a qualitative analysis of student reflections written during the final session of a Becoming a Physician curriculum."*	Qualitative study; narrative reflections	*"Narrative themes separated into four distinct categories, specifically that performing physician tasks can make one feel like a doctor, demonstrating caring is a fundamental task of doctors, integrating personal ideals with professional values promotes professional identity formation, and the theme of never feeling like a doctor. Subsets of these broad categories provide further insight into individual and integrative tasks. Patients, patient families, and students through their own reflection prompted learners to feel like doctors in 74% of narratives, whereas physicians or the care team did so in 26% of the narratives."*	*"Students are able to reflect on times during their principal clinical year where they feel like doctors, taking a step toward forming a professional identity. Having faculty prompt and support such reflection can help faculty understand the student experience of their principal clinical year and promote professional identity formation."*
**32**	Helmich et al.	Do you really want to be a doctor? The highs and lows of identity development	2012	*"The development of professional identity, or truly becoming a doctor, is central to medical students' education. How people develop that identity, learn to act in an appropriate way, and demonstrate confidence when doing so, receives less attention. This commentary sets out to do two things: firstly, to compare Burford's social identity perspective with an alternative theory, highlighting the relative differences and strengths of each, and,*	Commentary	*NIL*	*"Burford's illustration of how theory can illuminate research and practice and his exploration of professionalism and teamwork are valuable contributions. Likewise, his illustration of how belonging or not belonging to the in-group and perceiving or not perceiving a fit can have profound emotional and behavioural consequences is valuable in that it shows identification and identity development to be highly emotional processes. Although [the authors] support Burford in encouraging educators to question how accessibility and fit are salient*

				secondly, to explore in greater depth some of the issues Burford raises."			*in the experiences of medical students and new doctors, [the authors] urge them to be sensitive to the emotional highs as well as the lows of identity formation. [he authors] suggest that explicitly taking into account the emotional dimension of learning in workplaces will push the field an- other step forward and perhaps help more young people really want to be doctors."*
**33**	Holden et al.	Professional identity formation in medical education: the convergence of multiple domains	2012	*"There has been increasing emphasis on professionalism in medical education over the past several decades, initially focusing on bioethical principles, communication skills, and behaviors of medical students and practitioners."*	Perspective paper	*NIL*	*"The literature has approached professional identity formation from various paradigms—professionalism, psychological ego development, social interactions, and various learning theories. Similarities have been identified between the formation process of clergy and that of physicians. professional identity formation. Guided reflection, internalization, relationship building, the need for role models, the need for early experiences, and the processes of exploration and commitment are themes that appear throughout these interrelated domains." "Each perspective brings an important and unique lens to the multifaceted phenomenon of identity construction. More detailed explication of the process of professional identity formation in medical education should involve application of the principles and frameworks from these varied disciplines. The construction of professional identity in medical students is a complex, iterative process."*
**34**	Holden et al.	Professional identity formation: creating a longitudinal framework through TIME (Transformation in Medical Education)	2015	*"The University of Texas System established the Transformation in Medical Education (TIME) initiative to reconfigure and shorten medical education from college matriculation through medical school graduation. One of the key changes proposed as part of the TIME initiative was to begin emphasizing professional identity formation at the premedical level."*	Descriptive paper	*NIL*	*"In this article, [the authors] describe the task force's process for defining professional identity formation and developing a framework, which includes 10 key aspects, 6 domains, and 30 subdomains to characterize the complexity of physician identity. The task force mapped this framework onto three developmental phases of medical education typified by the undergraduate student, the clerkship-level medical student, and the graduating medical student. The task force provided strategies for the promotion and assessment of professional identity formation for each subdomain at each of the three phases, in addition to references and*

Appendix 6. _Tabulated Summaries_

							resources. Assessments were suggested for student feedback, curriculum evaluation, and theoretical development." "[The authors] emphasize the importance of longitudinal, formative assessment using a combination of existing assessment methods. Though not unique to the medical profession, professional identity formation is critical to the practice of exemplary medicine and the well-being of patients and physicians."
**35**	Iserson	Talking about professionalism through the lens of professional identity	2018	*"Professionalism is one of the Accreditation Council for Graduate Medical Education's (ACGME) Core Competencies, but the breadth of its content often makes this a difficult topic, both in remedial counseling and when presenting the topic to medical trainees and practicing clinicians. Physician professionalism encompasses both clinical competence and the virtues that comprise the physician's social contract. This difficult subject may best be approached tangentially, through the lens of professional identity."*	Commentary	*NIL*	*"Professional identity describes clinicians' affinity for, acculturation into, and identification with the practice of medicine. One method to highlight the benefits that individuals accrue by adopting professionalism's elements is to pose questions that optimize listeners' self-reflection about their lives and aspirations—in essence, their professional identity. Discussing professionalism this way often yields in-depth discussions of how trainees believe their professional identity was formed and will impact their long-term goals. Both in teaching and in counseling, educators can frame their discussions using professionalism and professional identity's overlapping and reinforcing elements to show listeners how to advance their personal and professional goals and avoid the short- and long-term consequences of unprofessional behavior. To engage the audience, educators and supervisors can emphasize how adhering to the elements of professionalism may determine their career opportunities, the professional respect they receive, and their career fulfilment and, ultimately, longevity. In this way, educators can better guide trainees and clinicians to understand their personal reasons for acting professionally, that is, doing the right thing, at the right time, in the right way, and for the right reason."*
**36**	Jarvis-Selinger et al.	Understanding professional identity formation in early clerkship: a novel framework	2019	*"Medical educators should foster students' professional attitudes because individuals are more likely to act in accordance with medicine's professional values if these values have been internalized. Still, there is much to be*	Qualitative study; collaborative discussions	*"Discussions facilitated students' reflection on their professional journeys. Analysis of transcribed discussions resulted in a conceptual framework useful for exploring and understanding students' reflections on their*	*"Discrete reflections focused on either students' current identity (being) or their sense of future self (becoming). The study identified catalysts that sparked participants' introspection about, or their processing of, identity. The moments that generate*

Appendix 6. Tabulated Summaries

				learned about how students examine and negotiate their emerging identities. This study examined third-year medical students' experiences of professional identity formation during clinical clerkship."		*professional identity formation. Through analyzing students' experiences, [the authors] identified four components that constituted professional identity formation stories: context, focus, catalyst, process."*	*profound feelings of awareness in students are often moments that would not be recognizable (even post hoc) as remarkable by others."*
**37**	Kalen et al.	Longitudinal mentorship to support the development of medical students' future professional role: a qualitative study	2015	*"Mentoring has been employed in medical education in recent years, but there is extensive variation in the published literature concerning the goals of mentoring and the role of the mentor. Therefore, there is still a need for a deeper understanding of the meaning of mentoring for medical students' learning and development. The aim of this qualitative study is to explore how formal and longitudinal mentoring can contribute to medical students' professional development. Medical students at a Swedish university were interviewed individually about their experiences of combined group and one-to-one mentoring that is given throughout their studies."*	Qualitative study; interviews	*"The results comprise three themes: integrating oneself with one's future role as a physician, experiencing clinical reality with the mentor creates incentives to learn and towards understanding the professional competence of a physician. The mentorship enabled the students to create a view of their future professional role and to integrate it with their own personalities. The students' understanding of professional competence and behaviour evolved during the mentorship and they made advances towards understanding the wholeness of the profession."*	*"Formalized and longitudinal mentoring focusing on the non-medical skills can be recommended to help medical students to integrate their professional role with themselves as individuals and promote understanding of professional competence in the process of becoming a physician."*
**38**	Kavas et al.	Turkish students' perceptions of professionalism at the beginning and at the end of medical education: a cross-sectional qualitative study	2015	*"Medical students' perceptions of professionalism might reflect the impact of the current educational processes on their professional identity development. This study focuses on Ankara University Faculty of Medicine students' perceptions of 'good doctor' along with the factors effective on the formation of these perceptions."*	Qualitative study; focus groups	*"The thematic pattern of the discussions on the relation between professional development and medical education suggests that students suffer from a gradual erosion of perception during medical education. That the education cannot either change the person for the better or might downgrade the person instead of improving her/him were shared by participants from both grades. Students consider clinical practice and role models two main variables determining the person's qualification as a professional."*	*"The formal and hidden programs determine the quality and efficacy of the professional education together. Attempts to restructure medical education must recognize the reciprocal dynamics between these two components and, thus, should carefully work out the practical aspect of the educational processes."*
**39**	Kirkpatrick et al.	Medical students are not blank slates: Positionality and curriculum interact to develop professional identity	2018	*"This commentary aims to reflect on how diverse medical student positionalities influence professional identity formation. Moreover, [the authors] aim to highlight the role of medical education institutions' formal and hidden curricula in empowering students to develop a professional identity that embraces their positionality."*	Commentary	*NIL*	*"While the medical education community is making great efforts to address this issue, [the authors] draw on their experiences as students to suggest three key approaches institutions can adopt to better facilitate professional identity formation by meaningfully embracing diversity, (1) access to student services, (2) near-peer and faculty mentorship, (3) formal curricula."*

							"In addition, empowering students to integrate their pre-medical backgrounds into their developing identity is crucial not only to improving the process of professional identity formation for diverse students, but also to nurturing empathic and insightful physicians uniquely suited to respond to the plights of their patients."
**40**	Konkin et al.	Creating stories to live by: caring and professional identity formation in a longitudinal integrated clerkship	2012	*"Building on other models of longitudinal integrated clerkships (LIC), the University of Alberta developed its Integrated Community Clerkship with guiding principles of continuity of care, preceptor and learning environment. This study explored the development of an ethic of caring in an LIC using empathy, compassion and taking responsibility as descriptors of caring."*	Qualitative phenomenological study; semi-structured reflective conversations	*"Continuity afforded by the LIC results in a safe environment in which students can meaningfully engage with patients and take responsibility for their care under the supervision of a physician teacher. Together these attributes foster an emerging physician identity born at the site of patient-student interaction and grounded in an ethic of caring."*	*"A medical student's evolving professional identity in the clerkship includes the emergence of an ethic of caring. Longitudinal integrated clerkships make this possible by providing time for relationships to develop with patients, preceptors and other health professionals."*
**41**	Lin	Fostering students' professional identity using critical incident technique	2012	*"Some professional development courses are currently provided, but the limitations of existing approaches include the fact that didactic methods are unhelpful to students' integration. Critical incident technique (CIT) is an in-depth investigative tool for multi-site case studies derived from qualitative grounded theory. It identifies the contexts, strategies and outcomes of emotion-laden critical events from which investigators form objective criteria for further application. The instructor utilised CIT as an observational framework to collect role models and analyse responses to critical incidents, facilitating students' professional identity development through narration."*	Short report of an interventional study; course evaluation questionnaires	*"Data sourced from final anonymous course evaluation questionnaires completed by 30 respondents showed that 77% of respondents ranked CIT speeches as the most effective teaching strategy. The main themes of students' reflective processes were: (i) doctors' descriptions of the nature of medicine interwoven with arts and science in approaches to patient difficulties and student needs; (ii) how doctors demonstrated compassion for human suffering by meeting patients' less obvious needs; (iii) how role models resolved conflicts between professionalism and personal or organisational interests, and (iv) contemporary doctors' values. A 6-month follow-up focus group interview found the same benefits."*	*"Students valued the first-hand accounts of experiences given in the doctors' lectures. Instructor comments and student reflections also deepened experiential learning about professionalism. Critical incident technique-based speeches by different specialists and students' reflective writing with facilitator feedback combined to make an effective approach towards group mentoring. While providing a useful observational framework with which to investigate relevant contexts and factors involved in emotionally loaded critical incidents, CIT enhanced students' understanding of their feelings and thoughts regarding their personal lives and future professional careers."*
**42**	Madill et al.	Identity change and the human dissection experience over the first year of medical training	2005	*"It is known that education and occupation are important influences on identity in young adults. The formation of professional identity has, however, rarely been studied in relation to medical students even though the ramifications of this process may be huge. The aim of this study is to explore identity change in medical students over their first year of medical*	Interventional study	*"The participants were optimistic about becoming similar to a doctor they admired and, towards the end of term three, began to develop a stable identity as a medical student. Their identity constructs involved three common themes: dedication, competence, and responsibility. However, the data also revealed negative reactions to the demands of training, such as feeling*	*"The participants' dedication to their studies was reflected in their appreciation of the need to become involved actively in the process of dissection but some experienced an erosion of their self-confidence and perceived some of their colleagues to have lost much of their enthusiasm for learning. [The authors] see a development of a vulnerable sense of professionalism alongside a frustration of losing out*

				training, particularly in relation to their experience of human dissection. Each of the four participants completed two repertory grids at the end of term one and, again, towards the end of term three. One grid tapped their identity construction, and the other, their experience of human dissection."		*driven and stressed. Three major themes were apparent in their experience of human dissection: involvement, emotional coping, and ability."*	*potentially on wider aspects of personal development due to the high work demands."*
**43**	Miller et al.	Sounding narrative medicine: studying students' professional identity development at columbia university college of physicians and surgeons	2014	*"At the College of Physicians and Surgeons of Columbia University (P&S), all students participate in training in narrative medicine (NM), a field with roots in humanities, creative writing, social sciences, the arts, and reflective clinical practice. The study aims to learn what medical students derive from training in humanities, social sciences, and the arts in a narrative medicine curriculum and to explore narrative medicine's framework as it relates to students' professional development. On completion of required intensive, half-semester narrative medicine seminars in 2010, second-year medical students at Columbia University College of Physicians and Surgeons participated in focus group discussions of their experiences."*	Qualitative study; focus group discussions	*"Students' comments articulated the known features of narrative medicine—attention, representation, and affiliation—and endorsed all three as being valuable to professional identity development. They spoke of the salience of their work in narrative medicine to medicine and medical education and its dividends of critical thinking, reflection, and pleasure. Critiques constituted a small percentage of the statements in each category."*	*"Students report that narrative medicine seminars support complex interior, interpersonal, perceptual, and expressive capacities. Students' lived experiences confirm some expectations of narrative medicine curricular planners while exposing fresh effects of such work to view."*
**44**	Monrouxe et al.	Between two worlds: medical students narrating identity tensions	2013	*"Medical education entails more than just learning knowledge and skills; it is also about the formation of a new identity: learning about the attitudes, values, and behaviors expected of a doctor. Medical students inevitably experience a number of stressful situations, including the complex process of negotiating personal and professional identities in their encounters with others. [The authors] focus on narratives from a longitudinal solicited audio diary study investigating how individuals entering medical education develop their identities as medical students and doctors through the spontaneous stories they conveyed over time."*	Qualitative study; narrative reflections	*"Medical students do not start on a clean slate when they are developing their professional identities. This may result in conflicts between existing and desired identities. The narratives represent the initial perspectives of looking onto, living alongside and living with illness, dying, and death, focusing on students' subsequent struggle between personal and professional identities."*	*"Bearing and/or reconciling two conflicting identities causes stress and confusion in medical students. Through such reflection students could consider how things might change in the future, both in their understandings of themselves and their understandings of interactions with others."*
**45**	Noguera et al.	Palliative care teaching shapes medical	2018	*"The aim of this review is to understand how palliative care teaching (PCT) as a patient-centered learning model,*	Review	*"Fifteen studies were selected: ten of those studies used a qualitative approach; two are theoretical*	*"PCT seems to be an effective way of fostering medical undergraduate students' patient-centered professional development."*

		undergraduate students' professional development: a scoping review		*influences medical undergraduate students' professional development. To study PCT medical undergraduate students' learning experiences, [the authors] employed the medical teaching concept, 'hidden curriculum,' as a way of describing attitudes and behavior conveyed implicitly by palliative care educators."*		*explanations of the topic explored, one guideline, one review and just one quantitative study, made up the review. Medical undergraduate students reported that after PCT, they felt they had acquired better attitudes for effective integration with the patient, such as empathy or holistic care; ethical principles, such as respect or humanization of their clinical practice; and commitment to an improvement in competences, such as self-awareness or self-esteem. They also reported improved behavior in effective integration with patients, such as communication, caring for patients' families, and when addressing psychosocial, cultural and spiritual aspects; their commitment to improvement in competences, such as dealing with emotions and uncertainty; they learned team work as an effective way to interact within the health system; and to become more reliable, making themselves more available and dedicating enough time to each patient."*	
**46**	O'Regan et al.	Social media and professional identity: Pitfalls and potential	2018	*"Social media developments have completely changed how information is accessed and communicated. While great potential exists with these platforms, recent reports of online unprofessional behavior by doctors has threatened the medical professional identity; a matter of critical importance for clinicians and medical educators. This paper outlines a role for social media in facilitating support for clinicians and medical teachers; it will raise awareness of pitfalls and explain ethical and legal guidelines."*	Perspective paper	*NIL*	*"It is essential that medical educational and professional bodies encourage clinicians to support one another and share information online while providing clear legal and ethical advice on maintaining standards and avoiding common pitfalls." "Education on the responsible use of social media and associated risk awareness should be a priority for medical school curricula."*
**47**	Olive & Abercrombie	Developing a physician's professional identity through medical education	2017	*"Professionalism represents a fundamental characteristic of physicians. Professional organizations have developed professionalism competencies for physicians and medical students."*	Review	*"Addressing lapses and critical reflection is an important part of the educational process. The "hidden curriculum" within an institution plays an important role in professional identity formation. Assessment of professionalism involves multiple mechanisms. Steps in remediating professionalism lapses include (1) initial assessment, (2)*	*"Professional role models, experiences from working on a team and moments for reflection with normative justification are key elements when designing teaching methods for professionalism. In addition, a significant relationship between a medical student's reflective ability and professionalism has been shown to exist. A wide variety of other instructional methods have been reported.*

						diagnosis of problems and development of an individualized learning plan, (3) instruction encompassing practice, feedback and reflection and (4) reassessment and certification of competence. No reliable outcomes data exist regarding the effectiveness of different remediation strategies."	*The placement of these instructional methods should be intentional throughout the curriculum, with sentinel events targeted for integration of professional identity formation opportunities. To ensure efforts are successful, faculty development is necessary to ensure a clear understanding of the nature of professionalism and the methods of teaching and assessment appropriate for the prescribed learning environment."*
**48**	Peterson et al.	Understanding the struggles to be a medical provider: view through medical student essays	2018	*"The clinical learning environment helps to shape the professional identity of medical students. This process begins from existing personal identity and is influenced by various factors, including clinical experiences and clinical learning environment. The purpose of this study was to examine medical students' reflections as a way to identify and better characterize the modern struggles that medical students face, in order to inform the development of professional identity. Students rotating in their emergency medicine clerkship wrote reflections on dilemmas that highlighted common struggles of becoming a doctor."*	Qualitative study; written reflections	*"The first domain was Patient–Provider Conflict, including challenging patient (34%), difficult communication (25%), competing priorities between patients' interest and trainees need to learn (19%), and bias (13%). The second domain was Provider-Specific Issues, such as the ''gray zone,'' in which there is not a clear standard of practice (29%), end-of-life care (14%), emotional struggle (6%), and fear of litigation (5%). The final domain was Systems Issues, such as cost of care (12%) and role of the emergency department (6%)."*	*"The reflections point to a wide variety of challenges that students confront in practice that will contribute to how they develop into physicians."*
**49**	Rabow et al.	Professional formation: extending medicine's lineage of service into the next century	2010	*"To meet the complex medical and social challenges of the next century, medical educators must continue to promote cognitive expertise while concurrently supporting "professional formation"—the moral and professional development of students, their ability to stay true to their personal service values and the core values of the profession, and the integration of their individual maturation with growth in clinical competency. The goal of professional formation is to anchor students to foundational principles while helping them navigate the inevitable moral conflicts in medical practice. The consequences of inadequate support for professional formation are profound, impacting individual learners, patients, the profession, and society at large."*	Perspective paper	*NIL*	*"Committing to professional formation within medical education will require transformation of formal and informal curricula and will necessitate a rebalancing of attention and financial support within schools of medicine."*<br><br>*"Professional formation education offers both students and faculty the support to make their foundational values the principles of action in daily life and to bring their whole selves to work. The personal rewards of healing the divided life are integrity, self-respect, and connection."*

Appendix 6. <u>Tabulated Summaries</u>

**50**	Rabow et al.	Authentic community as an educational strategy for advancing professionalism: A national evaluation of the Healer's Art course	2007	*"Efforts to promote medical professionalism often focus on cognitive and technical competencies, rather than professional identity, commitment, and values. The Healer's Art elective developed by Rachel Naomi Remen, M.D., at the Institute for the Study of Health and Illness (ISHI) at Commonweal is designed to create a genuine community of inquiry into these foundational elements of professionalism. Evaluations were obtained to characterize course impact and to understand students' conceptions of professionalism."*	Qualitative study; narrative reflections	*"From a question about what students learned about the practice of medicine from the Healer's Art, the most common themes were "definition of professionalism in medicine" and "legitimizing humanism in medicine." The most common themes produced by a question about the most valuable insights gained in the course were "relationship between physicians and patients" and "creating authentic community." The most common themes in response to a question about course utility were "creating authentic community" and 'filling a curricular gap'."*	*"In legitimizing humanistic elements of professionalism and creating a safe community, the Healer's Art enabled students to uncover the underlying values and meaning of their work—an opportunity not typically present in required curricula. Attempts to teach professionalism should address issues of emotional safety and authentic community as prerequisites to learning and professional affiliation."*
**51**	Reis et al.	Contemplating medicine during the Third Reich: scaffolding professional identity formation for medical students	2015	*"The moral failures of physicians and the medical establishment in Germany and Austria during the Third Reich challenge medicine and medical education in a way few other events do. They compel medical educators to ensure that lessons learned from contemplating medicine during the Third Reich be integrated into current and future physicians' professional identities. Most health professions education programs, however, have not adopted this study domain in their curricula. A new curriculum module - "The Holocaust and Medicine"- and its implementation in October 2013 at Bar-Ilan University Faculty of Medicine in the Galilee, Safed, Israel, as a requirement for all medical students (starting with the class of 2017) is outlined."*	Descriptive paper	*NIL*	*"The programme was described. This innovative module integrates historical facts, guided reflection, flipped classroom pedagogy, and program evaluation efforts. It spans 20 months of the preclinical curriculum, embedded within a doctoring course and a medical humanities longitudinal course and integrated within the clinical sciences blocks." "[The authors] propose that contemplation of medicine after the Holocaust and the implications for contemporary practice should be an integral component of health professions education to promote humanistic, ethically responsible practice."*
**52**	Roper et al.	The challenge of authenticity for medical students	2016	*"The development of a professional identity occurs during medical school. Formal study of students' reflections on this process may provide insight into how to better support them."*	Qualitative study; narrative reflections	*"Students' early interactions with patients seem to be influential in their process of identity development. Students were preoccupied with creating or preserving a professional persona in front of patients. They responded to this perceived challenge in three ways: some were concerned with controlling the experience and expression of emotion, others felt that they failed to be authentic, and the third group focused on the patient's experience of the interaction and*	*"This article adds to the literature by highlighting the struggles medical students encounter trying to behave and feel the way they think they ought. Students may be less troubled and participate more naturally in empathic communication if they learn to access authentic emotions in their interactions with patients. This article discusses strategies for medical faculties and clinical tutors to support and encourage them to do so. Students' early interactions with patients seem to be influential in their process of identity development."*

Appendix 6. <u>*Tabulated Summaries*</u>

						agonised over what the patients might want."	
**53**	Schei et al.	Comfort zone: using early clinical contact to influence professional identity formation in medical students	2019	*"Early patient encounters create complex emotional challenges and constitute fertile ground for professional identity formation. The literature indicates that students often learn, largely through the hidden curriculum, to avoid and suppress emotion. This can culminate in mental health problems and loss of empathy. At the University of Bergen, a structured course in patient contact, PASKON, was created in 2013, with the double aim of helping first-year medical students learn from patients how sickness affects life and of guiding them toward patient-centered skills and an empathic presence in encounters with severely ill persons. The aim of this study was to explore how students described their thoughts, emotions, and behavior in essays written shortly after their first professional encounter with a gravely ill person, in the person's home."*	Qualitative study; reflective essays	*"Students described a wide range of affect-laden responses, positive and negative, elicited by the home visits. The observations were typically related to loss of control, struggles to behave "professionally," and the unmasking of stereotypes and prejudices."*	*"Medical students' initial clinical encounters elicit emotional responses that have the potential to serve as triggers for the development of emotional maturity, relational skills, and patient-centered attitudes. Conversely, they can foreground uncertainty and lead to defensive distancing from patients' existential concerns. The findings point to a role for structured educational strategies and supervision to assist students in the emotion work necessary in the transition from a "lay" to a "medical" identity."*
**54**	Schei et al.	Trustingly bewildered. How first-year medical students make sense of their learning experience in a traditional, preclinical curriculum	2018	*"Traditional preclinical curricula based on memorization of scientific facts constitute learning environments which may negatively influence both factual understanding and professional identity development in medical students. Little is known of how students themselves experience and interpret such educational milieus."*	Qualitative study; focus group interviews	*"Students portrayed the good physician as communicative, humble, and open, combining biomedical knowledge and moral strength. When asked how medical school supported the development of such characteristics, two partly contradictory discourses emerged. The critical discourse identified decontextualized knowledge, poor pedagogy, lack of critical thinking, and contact with faculty. Students who voiced critical comments also articulated trust that the system would provide the competence they needed, that basic biological knowledge is needed before clinical practice, and that being on your own conveys freedom and responsibility, and helps you grow up".*	*"Trust in the educational system, within a substandard learning environment, created cognitive dissonance that students resolved through rationalization, whereby they negated that factual overload and lack of relevance, reflection, and personal feedback was problematic. The cost of this mechanism is possibly that inferior teaching is perceived as normal, necessary, and good enough. If so, these future physicians' ability to critically evaluate and create quality in medical education and practice, may be weakened."*
**55**	Schrewe et al.	The Big D(eal): professional identity through discursive constructions of 'patient'	2017	*"Professional identity formation has become a key focus for medical education. Who one becomes as a physician is contingent upon learning to conceptualise who the other is as a patient, yet, at a time when influential*	Qualitative study; narrative reflections	*"Identified discourses included patient-as-disease-category, patient-as-educational-commodity and patient-as-marginalised-actor. These discourses conceptualise 'patient' as an entity that is principally biomedical, useful for*	*"[The authors] contend that as learners participate in these discourses, they are also performatively produced by them. By making these discourses visible, we can consider how to minimise unintended effects such discourses may cause. The*

Appendix 6. Tabulated Summaries

				ideologies such as patient-centred care have become espoused values, there has been little empirical investigation into assumptions of 'patient' that trainees take up as they progress through their training. The team employed a critical discourse analysis approach to transcripts originally produced from a micro-ethnography of medical student learning on an acute care in-patient paediatric ward. [The authors] paid specific attention to how trainees used language to talk about, refer to and categorise patients."		*clinical learning and spoken for and about. Medical student participation in these discourses contributes to an identity that allows them to move further into the professional medical world they are joining."*	*findings, although limited, offer a glimpse of the effects that those assumptions may have as we look to align better the formation of professional medical identity with the ideals of patient-centred care and socially responsible health care systems."*
**56**	Schweller et al.	Nurturing virtues of the medical profession: does it enhance medical students' empathy?	2017	*"The study aims to examine if the empathy levels of first-year medical students are amenable to didactic interventions idealized to promote values inherent to medical professional identity. This is a pretest-posttest study designed to assess the empathy levels of first-year medical students comprising two consecutive classes of a Brazilian medical school, performed before and after a didactic intervention. Students attended a course based on values and virtues related to medical professional identity once a week over four months. Every didactic approach (interviews with patients and physicians, supervised visits to the hospital, and discussion of videotaped simulated consultations) was based on "real-world" situations and designed to promote awareness of the process of socialization."*	"Interventional study; students filled out the Jefferson Scale of Physician Empathy (JSPE) on the first and last days of this course, and the pretest-posttest analysis was performed using the Wilcoxon Signed Rank Test"	*"The mean pretest JSPE score was 117.9 (minimum 92, maximum 135) and increased to 121.3 after the intervention (minimum 101, maximum 137). The difference was significant (z=-5.2, p<.001.), with an effect size of 0.40. The observed increase was greater among students with lower initial JSPE scores."*	*"Empathy is a fundamental tool used to achieve a successful physician-patient relationship, and it seems to permeate other virtues of a good physician. This study's results suggest that medical students' empathy may be amenable to early curricular interventions designed to promote a positive development of their professional identity, even when empathy is not central in discussion."*
**57**	Seymour et al.	Professional Competencies ToolKit: using flash cards to teach reflective practice to medical students in	2018	*"Early clinical experiences can be overwhelming to medical students. The Professional Competencies ToolKit (ProComp ToolKit) gives medical students a framework on which to build these early experiences and reflect on issues related to professionalism as each patient encounter unfolds."*	Interventional study, qualitative analysis of student narratives	*"[The authors] demonstrated how the student narratives that emerged from using the flash cards, exchanged in a small-group setting, led to group problem solving and validation of students' experiences and values. In the narratives, students discussed the origin of negative behaviors and attitudes that can become normalized in patient care while asserting the primacy of patient-centered care and devising self-awareness strategies."*	*"[The authors]' experience using the ProComp ToolKit shows that teaching reflective practice can successfully be integrated into students' clinical experiences. Professionalism skills can be reflected upon such that they become habitual and integral to students' developing professional identities."*
**58**	Shapiro et al.	Medical students' efforts to	2018	*"Medical students' mask-making can provide valuable insights into personal*	Interventional study,	*The analysis of individual masks and narratives revealed many dimensions*	*"This grand theme acknowledged that, in students' views, identity does not consist of*

		integrate and/or reclaim authentic identity: Insights from a mask-making exercise		*and professional identity formation and wellness. A subset of first- and second-year medical students attending a medical school wellness retreat participated in a mask-making workshop."*	examination of student masks and explanatory narratives	*such as topics and concerns, sectionality, salience, tone, emotions, self, needs, wellness. [The authors] identified an overarching theme: "Reconciliation/reclamation of authentic identity."*	*just one element but rather reflects many different aspects of self. The combination of nonverbal mask-making and narrative offers rich insights into medical students' experience and thinking. This activity promoted reflection and self-care, while providing insight regarding personal and professional development."*
**59**	Sharpless et al.	The Becoming: students' reflections on the process of professional identity formation in medical education	2015	*"Professional identity formation within medical education is the multifaceted, individualized process through which students develop new ways of being in becoming physicians. Personal backgrounds, values, expectations, interests, goals, relationships, and role models can all influence professional identity formation and may account for diversity of both experience and the active constructive process of professional formation. This work aims to vividly illustrate the diverse and personal forces at play in individual students' professional identity formation processes and to encourage future pedagogic efforts supporting healthy, integrated professional identity formation in medical education. In this commentary, [the authors] present a chorus of individual student voices from along the medical education trajectory. Medical students (years 1–4) and a first-year resident in pediatrics respond to a variety of questions based on prevalent professional identity formation themes extracted from the literature to reflect on their personal experiences of professional identity formation."*	Commentary	*NIL*	*"Topics queried included pretending in medical education, role of relationships, impact of formal and informal curricula on professional identity formation (valuable aspects as well as suggestions for change), and navigating and developing interprofessional relationships and identities."*
**60**	Shiozawa et al.	An insight into professional identity formation: qualitative analyses of two reflection interventions during the dissection course	2019	*"The professional behavior of future doctors is increasingly important in medical education. One of the first subjects in the curriculum to address this issue is gross anatomy. The Tuebingen Medical Faculty implemented a learning portfolio and a seminar on medical professionalism during the dissection course. The aims of this research project are to get an overview of how students form a professional identity in the dissection*	Qualitative study; oral and written reflections on the dissection laboratory experience	*"Both qualitative analyses show that students reflected on many topics relevant to professional development, including empathy, respect, altruism, compassion, teamwork, and self-regulation. Quantitative analysis reveals that students who attended the oral reflection wrote significantly more in their written reflection than students who did not. There is, however, no difference in the reflection categories. Reflection content from students corresponds with*	*"Both the seminar (oral reflections) and the learning portfolio (written reflections) present excellent opportunities to foster professional development during anatomy education; the key is using them in conjunction with the dissection course."*

				course and to compare the content of both their oral and written reflections on the course."		*categories derived from existing competency frameworks."*	
**61**	Sklar	How do I figure out what I want to do if I don't know who I am supposed to be?	2015	*"In an opening letter, the then editor of Academic Medicine offers personal anecdotes reiterating the importance of professional identity formation and outlines various articles in the issue which discuss the concept in greater scope and depth."*	Editorial	*NIL*	*"[The authors] in this issue describe programs, teachers, and the educational environment that create opportunities for personal and professional growth for trainees. These authors suggest that in the development of a medical student, programs in professional identity formation can be equal partners with programs that emphasize acquisition of procedural skills and medical knowledge."*
**62**	Slotnick	How doctors learn: education and learning across the medical-school-to-practice trajectory	2001	*"Doctors begin developing the skills and knowledge they need in medical school and continue developing them through residency and into practice. However, there are important differences among medical students, residents, and practicing physicians, and these differences have an impact on how the individuals satisfy their psychosocial needs. These differences also bear on how well they will satisfy their needs later in their careers; i.e., the things physicians-in-training do to satisfy their psychosocial needs in the course of learning to become doctors are expected to prepare them to address their psychosocial needs later in life in their roles as both healers and professionals. It is against this background of describing how education helps physicians solve problems and so address their recurring psychosocial needs that [the author] presents twelve conclusions concerning physicians' learning."*	Commentary	*NIL*	*"(1) While the goal of medical education is to produce physicians who will work within a health care environment in the future, the education mechanisms employed develop identities within physicians-in-training, allowing them to address their immediate needs. More specifically, they learn whatever they need to satisfy immediate needs while developing the knowledge and skills necessarily insufficient to address their psychosocial needs at subsequent points along the medical school-through-practice trajectory.<br>(2) Experience in the clinical practice of medicine is necessary before physicians can incorporate what they have learned in medical school and residency into the bodies of skill, knowledge, and experience they use in satisfying their psychosocial needs by caring for patients.<br>(3) The instructional activities that engage and maintain the attention of physicians-in-training and physicians have impacts on their identities and thus their abilities to address their security, affiliation, and self-esteem needs.<br>(4) Expectations held by people with whom physicians-in-training and physicians interact effectively define what the physicians-in-training and the physicians must do to satisfy their Maslowian psychosocial needs.<br>(5) Knowledge and skills learned early in a physician-in-training's medical school-through-practice trajectory must be revisited*

							(relearned) at subsequent points in the trajectory so that what has been learned under an earlier set of expectations can be reconsidered in light of newer, more sophisticated expectations.<br>*(6) Because of psychosocial changes that affect doctors' lives, it is inappropriate to certify [1] physicians in a given cohort in the same ways when they complete medical school, residency, and later become practicing physicians, since the certification is not attuned to the related stage of identity development each physician has reached; and [2] different cohorts at the same points in their careers (e.g., at the completion of residency training) unless normative age-graded expectations are unchanged and non-normative history-graded matters remain the same. Instead, proper certification should consider social factors and the nature of the doctors' practices and the particular medical experiences they have had, psychosocial development, and epistemologic sophistication, as well as changes in biomedical theory and technology of medicine.*<br>*(7) While the medical profession claims ownership of a body of medical skills and knowledge, any given physician is knowledgeable about and has a perspective for accessing only a part of it, both the part and the perspective reflecting normative and non-normative influences on the doctor's life.*<br>*(8) In contrast to the knowledge of and perspective on the body of medical skill and knowledge, which varies from doctor to doctor, all doctors use the skills and knowledge to satisfy their Maslowian psychosocial needs through addressing medical problems for society.*<br>*(9) Even though the profession of medicine claims a body of esoteric, scientific information as its own, and that it uses that body of information to address medical problems for society, individual clinicians have a grasp of the corpus and an approach to it that are more practical, less scientific.*

							(10) Proper resolution of questions of conflict of interest requires an awareness of the needs of the individual physician, the medical profession, and society more generally.<br>(11) Being a skilled teacher of adults is a professional responsibility for physicians.<br>(12) The physician-as-healer and the physician-as-professional are two aspects of the physician's identity that evolve in tandem and in response to normative age-graded and history-graded influences."
**63**	Smith et al.	The effects of contributing to patient care on medical students' workplace learning	2013	*"Previous research has suggested that as medical students become more senior, they should increasingly take on the roles they will enact as newly qualified doctors by contributing to patient care. However, student contribution to patient care carries inherent risks to patient safety. This study aimed to provide students with a new opportunity to contribute to patient care and to use this as a platform from which to explore the influence of contributing to patient care on medical student learning. This study took place in the context of final-year medical student prescribing education at the University of Edinburgh, UK. Students on attachment at a district general hospital were afforded a unique opportunity to learn prescribing by completing in-patient drug charts in a process termed 'pre-prescribing'."*	Qualitative study; focus group discussions	*"The emerging themes took the form of developmental outcomes and learning processes. Developmental outcomes included ability to perform the task, modification of attitudes towards the task, formation of a professional identity, and development of relationships within the team. The central feature of the experience which influenced all developmental outcomes, was making mistakes. The themes interact in complex ways and all contribute towards development as a professional."*	*"This study has demonstrated that contributing to patient care enhances students' development as professionals. Some of these developmental outcomes, such as improvements in knowledge and skills, may be achievable to some extent within the classroom. Other changes, such as developing relationships, forming a sense of professional identity and modifying attitudes, might arguably be achievable only within the context of contributing to patient care."*
**64**	Soo et al.	At the precipice: A prospective exploration of medical students' expectations of the pre-clerkship to clerkship transition	2016	*"Medical learners face many challenging transitions. [The authors] prospectively explored students' perceptions of their upcoming transition to clerkship and their future professional selves."*	Mixed methods study; written narrative reflections and questionnaires	*"Two overarching themes were identified: (1) "Looking back": experiences which had helped students feel prepared for clerkship with subthemes focused on of patient care, shadowing, classroom teaching and the pre-clerkship years as foundational knowledge, (2) "Looking forward": anticipating the clerkship experience and the journey of becoming a physician with subthemes focused on death and dying, hierarchy, work-life balance, interactions with patients, concerns about competency and career choice. Questionnaire data revealed*	*"[The authors] confirmed that internal transformations are happening in contemplative time even before clerkship. By prospectively exploring pre-clerkship students' perceptions of the transition to clerkship training they identified expectations and misconceptions that could be addressed with future curricular interventions. While students are aware of and anticipating their learning needs it is not as clear that they realise how much their future learning will depend on their own inner resources. [The authors] suggest that more attention be paid to professional identity formation and the development of*

						incongruities around expectations of minimal exposure to death and dying, little need for independent study and limited direct patient responsibility."	*the physician as a person during these critical transitions."*
**65**	Steinauer et al.	What makes difficult patients" difficult for medical students?"	2018	*"Physicians can find it challenging to provide high-quality care to "difficult patients." While studies support that medical students also find some patients "difficult," little is known about why they do or how being a student affects their perceptions. [The authors] conducted this study to gain a deeper understanding of students' experiences with "difficult patients" to inform clinical teaching about effective patient communication and patient-centered care. In 2016, [the authors] conducted interviews with fourth-year medical students, who were asked to describe patient interactions in which they felt negative emotions toward the patient, as well as describe the clinical setting and their feelings."*	Qualitative study; interviews	*"Students described negative feelings toward patients and patients' behaviors, which were exacerbated by three situations related to their role and expectations as learners: (1) patients' interference with students' ability to "shine"; (2) patients' interference with students' expectations of patient-centered care; and (3) students' lack of the tools or authority to improve patients' health."*	*"Educators should consider these findings, which can be explained by the professional identity formation and goal orientation theory frameworks, as they teach medical students to provide high-quality care for patients they find 'difficult'."*
**66**	Stephens et al.	Examining professional identity formation through the ancient art of mask-making	2019	*"Professional identity formation is a complex sociocultural process whereby medical students learn to think, feel, and act like physicians. This process is often unscripted and influenced by informal curricular elements. Hypothesizing that a struggle in the process of identity formation is associated, [the authors] incorporate visual strategies to (re)introduce mask-making as a mechanism to foster professional identity formation in the context of medical education."*	Interventional study, qualitative analysis through visual rhetoric, a listening guide to analyze the visual components of each mask and accompanying written narratives	*"[The authors] identified themes of role strain, isolation, burnout, and identity dissonance to be the most common in medical student masks. Although potentially limited by scope and generalizability, the findings suggest that the students are able to reflect deeply on personal and professional identity-related issues and ex- press these through the mask-making experience."*	*"Recognizing that individuals view their present selves differently from their past or future selves, [the authors] feel that mask-making represents a unique way to longitudinally examine identity formation. Professional identity formation occurs at a pace that is specific to each student's experiences and personal contextual factors. Mask-making adds to the process of professional identity formation as a unique form of reflective expression using elements of artistry and non- linguistic expression. The expressive opportunities afforded by mask-making may provide additional insights into identity that are hard to capture in words, lending new direction to the scholarship of professional identity formation."*
**67**	Swick et al.	Fostering the professional development of medical students	1991	*"Medical education in the 1990s faces many challenges as it prepares graduates to practice in a rapidly changing environment. Recognizing that biomedical knowledge and skills alone are not sufficient preparation for medical practice, the Medical College of Wisconsin has implemented a 2-year*	Interventional study; quantitative analysis of Measure of Intellectual Development (MID) results	*"Results of a 3-year study designed to evaluate the impact of POMP on students' assumptions about the nature of knowledge and of uncertainty in medicine indicate that POMP students score significantly higher on the MID than do a non-equivalent control group of medical students."*	*"Educators should facilitate medical students' development of their professional identities, with an emphasis on the ability to make reasoned judgments in the face of uncertainty."*

				curriculum. Grounded in student development theory, the Profession of Medicine Program (POMP) is structured to challenge medical students' conceptions of the physicians' roles, responsibilities, values, and competencies through a series of short didactic courses and small-group preceptor meetings."			
**68**	ten Cate	What is a 21st-century doctor? rethinking the significance of the medical degree	2014	*"The undergraduate medical degree, leading to a license to practice, has traditionally been the defining professional milestone of the physician. Developments in health care and medical education and training, however, have changed the significance of the medical degree in the continuum of education toward clinical practice."*	Commentary	*NIL*	*"[The author] discusses six questions that should lead us to rethink the current status and significance of the medical degree and, consequently, that of the physician. These questions include the quest for core knowledge and competence of the doctor, the place of the degree in the education continuum, the increasing length of training, the sharing of health care tasks with other professionals, and the nature of professional identity in a multitasking world. [The author] concludes by examining ways to redefine what it means to be a 'medical doctor'"."*
**69**	ten Cate et al.	Time-variable training in medicine: theoretical considerations	2018	*"The introduction of competency-based medical education has shifted thinking from a fixed-time model to one stressing attained competencies, independent of the time needed to arrive at those competencies. In this article, [the authors] explore theoretical and conceptual issues related to time variability in medical training, starting with the Carroll model from the 1960s that put time in the equation of learning. They discuss mastery learning, deliberate practice, and learning curves."*	Perspective paper	*NIL*	*"While such behaviorist theories apply well to structured courses and highly structured training settings, learning in the clinical workplace is not well captured in such theories or in the model that Carroll proposed. Important in clinical training are self-regulation and motivation; neurocognitive perspectives of time and learning; professional identity formation; and entrustment as an objective of training-all of which may be viewed from the perspective of the time needed to complete training." "[The authors] conclude that, in approaching time variability, the Carroll equation is too simplistic in its application to the breadth of medical training. The equation may be expanded to include variables that determine effective workplace learning, but future work will need to examine the validity of these additional factors."*
**70**	Till et al.	Twelve tips for integrating leadership development into undergraduate	2018	*"Healthcare systems need effective leadership. All healthcare professionals can and should "learn to lead" and this requires a clear focus on leadership development from the earliest stages of*	Perspective paper	*NIL*	*"Drawing from the existing evidence base, [the authors]' expertise and the latest "thought leadership", these 12 tips provide practical guidance to universities and associated provider organizations, and to*

		medical education		*a career. Within medicine, undergraduate students should be provided with opportunities to thrive and develop their skills in terms of leadership, management and followership."*			*academic and clinical faculty, on how to integrate leadership development into their undergraduate medical programs. These 12 tips will help educators provide medical education that incorporates leadership as a core part of a professional's identity, and help students gain a deeper understanding of themselves and the teams, organizations and system they work within."*
**71**	van der Zwet et al.	Workplace learning from a socio-cultural perspective: creating developmental space during the general practice clerkship	2011	*"Workplace learning in undergraduate medical education has predominantly been studied from a cognitive perspective, despite its complex contextual characteristics, which influence medical students' learning experiences in such a way that explanation in terms of knowledge, skills, attitudes and single determinants of instructiveness is unlikely to suffice. There is also a paucity of research which, from a perspective other than the cognitive or descriptive one, investigates student learning in general practice settings, which are often characterised as powerful learning environments. In this study [the authors] took a socio-cultural perspective to clarify how students learn during a general practice clerkship and to construct a conceptual framework that captures this type of learning."*	Qualitative study; group interviews	*"Analysis of group interviews with fifth-year undergraduate medical students about their learning experiences in general practice showed that students needed developmental space to be able to learn and develop their professional identity."*	*"This space results from the intertwinement of workplace context, personal and professional interactions and emotions such as feeling respected and self-confident. These forces framed students' participation in patient consultations, conversations with supervisors about consultations and students' observation of supervisors, thereby determining the opportunities afforded to students to mind their learning."*
**72**	Volpe et al.	Does pre-clerkship medical humanities curriculum support professional identity formation? early insights from a qualitative study	2019	*"There is a lack of consensus about the outcomes of medical humanities training. Varied definitions suggest that there is a lack of consensus in the field about the most basic question: what is the fundamental nature of medical humanities learning? To begin to address this question, [the authors] conducted an exploratory study to analyze medical students' humanities small group discussions during the pre-clerkship period of their training at Penn State College of Medicine."*	Qualitative study; small group discussions	*"It quickly became apparent that the data set was steeped in the concept of professional identity formation. Three inter-related themes were identified: (1) students' perception/understanding of medicine as a profession, (2) students struggling with and reflecting on alignment of personal values and priorities with those of the profession, and (3) students grappling with ambiguity."*	*"The questioning, exploring, and searching that students do in medical humanities coursework help them explore what kind of physician they aspire to become. This exploration and subsequent articulation of values, preferences, and goals for the future sets the stage for integration of personal and professional identities—for professional identity formation. The study suggests that medical humanities small groups may support student colleagues in their difficult task of becoming."*
**73**	Wald et al.	Grappling with complexity: Medical students' reflective writings about challenging	2019	*"Clerkship-specific interactive reflective writing (IRW)-enhanced reflection may enhance professional identity formation professional identity formation, a fundamental goal of medical education.*	Immersion-crystallization qualitative analysis;	*"The qualitative analysis identified 26 unique emergent themes and five distinct thematic categories (1. Role of emotions, 2. Role of cognition, 3. Behaviorally responding to situational*	*"IRW facilitates and ideally supports grappling with the lived reality of medicine; uncovering a "positive hidden curriculum" within medical education. [The authors] propose engaging learners in guided critical*

		patient encounters as a window into professional identity formation		*Professional identity formation process as revealed in students' reflective writing (RW) has been understudied. [The authors] developed an IRW curriculum within a Family Medicine Clerkship (FMC) and analyzed students' reflections about challenging/difficult patient encounters"*	written reflections	*context, 4. Patient factors, and 5. External factors) as well as an emergent professional identity formation model from a directed content analysis. The model describes students' backgrounds, emotions and previous experiences in medicine merging with external factors and processed during student-patient interactions. The RWs also revealed that processing often involves polarities (e.g. empathy/lack of empathy or encouragement/disillusionment) as well as dissonance between idealized visions and lived reality first."*	*reflection about complex experiences for meaning-making within a safe learning climate as a valuable way to cultivate reflective, resilient professionals with "prepared" minds and hearts for inevitable challenges of healthcare practice."*
**74**	Wang et al.	Medical students' experiences with goals of care discussions and their impact on professional identity formation	2019	*"Goals of care (GoC) discussions occur amongst patients, family members and clinicians in order to establish plans of care and are invaluable aspects of end-of-life care. In previous research, medical learners have reported insufficient training and emotional distress about end-of-life decision making, but most studies have focused on postgraduate trainees and have been quantitative or have evaluated specific educational interventions. None have qualitatively explored medical students' experiences with GoC discussions, their perceptions of associated hidden curricula, and the impacts of these on professional identity formation the individualised developmental processes by which laypersons evolve to think, act and feel like, and ultimately become, medical professionals. Using purposive sampling at one Canadian medical school, individual semi-structured interviews were conducted with medical students to explore their experiences with GoC discussions during their core internal medicine clerkship"*	Qualitative study; semi-structured interviews	*"Participants reported minimal support and supervision in conducting GoC discussions, which were experienced as ethically challenging, emotionally powerful encounters exemplifying tensions between formal and hidden curricula. Role modelling and institutional culture were key mechanisms through which hidden curricula were transmitted, subverting formal curricula in doing so and contributing to participants' emotional distress. Participants' coping responses were generally negative and included symptoms of burnout, the pursuit of standardization, rationalization, compartmentalization and the adaptation of previously held, more idealised professional identities."*	*"Improved education about GoC discussions is necessary for patient care and may represent concrete and specific opportunities to influence students' on professional identity formation positively."*
**75**	Wilson et al.	Professional identity in medical students: pedagogical challenges to medical education	2014	*"Professional identity, or how a doctor thinks of himself or herself as a doctor, is considered to be as critical to medical education as the acquisition of skills and knowledge relevant to patient care. This article examines contemporary literature*	Review	*"This review uncovered the theoretical uniqueness of professional identity relative to professionalism in medicine. Further, the influence of pre-existing values and their role in the formation of professional identity in medicine was reiterated. Socialization and the*	*"[The authors] conclude that medical education needs to be responsive to changes in professional identity being generated from factors within medical student experiences or within contemporary society."*

				on the development of professional identity within medicine."		*influence of communities of practice, clinical settings, and patient and other health professional interactions were explored. The use of role models as a key pedagogical tool within medical curricula has been challenged, and the inclusion of such techniques as narrative reflection to enhance identity formation was posed. The role of technological change and consumer expectations in making demands upon the evolving professional identity of medical students has been outlined."*	
**76**	Wong et al.	Reflections: an inquiry into medical students' professional identity formation	2014	*"Professional identity formation plays a crucial role in the transition from medical student to doctor. At McMaster University, medical students maintain a portfolio of narrative reflections of their experiences, which provides for a rich source of data into their professional development. The purpose of this study was to understand the major influences on medical students' professional identity formation."*	Qualitative study; reflections within portfolios	*"Five major themes were associated with professional identity formation in medical students: prior experiences, role models, patient encounters, curriculum (formal and hidden) and societal expectations. The longitudinal analysis shows how these themes interact and shape pivotal moments, as well as the iterative nature of professional identity from the multiple ways in which individuals construct meaning from interactions with their environments."*	*"The study provides a window on the dynamic, discursive and constructed nature of professional identity formation. The five key themes associated with professional identity formation provide strategic opportunities to enable positive development. This study also illustrates the power of reflective writing for students and tutors in the professional identity formation process."*
